# Dynamic Cross Talk Model of the Epithelial Innate Immune Response to Double-Stranded RNA Stimulation: Coordinated Dynamics Emerging from Cell-Level Noise

**DOI:** 10.1371/journal.pone.0093396

**Published:** 2014-04-07

**Authors:** Roberto Bertolusso, Bing Tian, Yingxin Zhao, Leoncio Vergara, Aqeeb Sabree, Marta Iwanaszko, Tomasz Lipniacki, Allan R. Brasier, Marek Kimmel

**Affiliations:** 1 Department of Statistics, Rice University, Houston, Texas, United States of America; 2 Department of Internal Medicine, University of Texas Medical Branch (UTMB), Galveston, Texas, United States of America; 3 Sealy Center for Molecular Medicine, UTMB, Galveston, Texas, United States of America; 4 Institute for Translational Sciences, UTMB, Galveston, Texas, United States of America; 5 Center for Biomedical Engineering, UTMB, Galveston, Texas, United States of America; 6 Systems Engineering Group, Silesian University of Technology, Gliwice, Poland; 7 Institute of Fundamental Technological Research, Polish Academy of Sciences, Warsaw, Poland; Institut Pasteur, France

## Abstract

We present an integrated dynamical cross-talk model of the epithelial innate immune reponse (IIR) incorporating RIG-I and TLR3 as the two major pattern recognition receptors (PRR) converging on the RelA and IRF3 transcriptional effectors. bioPN simulations reproduce biologically relevant gene-and protein abundance measurements in response to time course, gene silencing and dose-response perturbations both at the population and single cell level. Our computational predictions suggest that RelA and IRF3 are under auto- and cross-regulation. We predict, and confirm experimentally, that RIG-I mRNA expression is controlled by IRF7. We also predict the existence of a TLR3-dependent, IRF3-independent transcription factor (or factors) that control(s) expression of MAVS, IRF3 and members of the IKK family. Our model confirms the observed dsRNA dose-dependence of oscillatory patterns in single cells, with periods of 1–3 hr. Model fitting to time series, matched by knockdown data suggests that the NF-κB module operates in a different regime (with different coefficient values) than in the TNFα-stimulation experiments. In future studies, this model will serve as a foundation for identification of virus-encoded IIR antagonists and examination of stochastic effects of viral replication.

Our model generates simulated time series, which reproduce the noisy oscillatory patterns of activity (with 1–3 hour period) observed in individual cells. Our work supports the hypothesis that the IIR is a phenomenon that emerged by evolution despite highly variable responses at an individual cell level.

## Introduction

The focus of this paper is to understand the dynamics of interaction between two major signaling pathways in the innate immune response (IIR) controlled by the nuclear factor-κB (NF-κB) and interferon response factor (IRF)-3 transcription factors that mediate inflammation and antiviral responses, respectively. The IIR is a signaling mechanism designed to limit the spread of infecting pathogen at mucosal surfaces before the adaptive immune response is activated [1]. The presence of “foreign” pathogen-associated molecular patterns, such as dsRNA and lipopolysaccharide, is recognized by a family of pattern recognition receptors (PRRs) that subsequently trigger signal transduction cascades. These cascades include the NF-κB and IRF transcription factors (TFs) [2], [3]. The link to adaptive immune protection is conferred by the expression of cytokine and protective interferons downstream of the NF-κB and IRF pathways. Interestingly, the intracellular IIR is not mediated by second messengers, but instead by signaling complexes produced by intracellular adapter molecules. These enzymes perform the functions of ubiquitylation, serine/threonine phosphorylation, and cysteinyl oxidation cascades that release and activate cytoplasmic TF complexes to enter the nucleus. Despite the finding that this pathway is activated in a robust manner, it is under very tight negative-feedback control [4], [5]. The properties of negative feedback of this system have been modeled using deterministic ordinary differential equations to understand the roles of negative feedback of inducible IκB-α, -β and -ε isoforms in regulating the temporal control of NF-κB [6], and our studies have modeled the roles of the NF-κB -TNFAIP3 feedback loop [7], [8]. Not much is known about how the activation of these two major signaling arms of the IIR is controlled. Recent work by our group and others has shown that adapter molecules regulating the IRF3 signaling pathway are inter-connected with those of NF-κB at multiple stages, with the final shared component being the IκB kinase-γ (IKKγ) subunit [9], [10]. More recently, single-cell imaging experiments have provided informative approaches to understanding the sources of cellular heterogeneity [11], [12]. Despite these and other experimental and modeling attempts, little has been known about how the NF-κB and IRF3 pathways interact with each other.

In addition to its tight control by intracellular negative cross-talk pathways, a full understanding of the IIR must incorporate cell-type dependent differences. For example, the patterns of IIR induced genes, their magnitude of induction and qualitative changes are different between epithelial cells and other cells of the innate pathway. These differences are due, in part, to the result of cell-type dependent expression and localization of key regulatory molecules. For example, in contrast to the cell-surface localization of TLR3 on monocyte/macrophages, TLR3 expression is endosomal in epithelial cells [13]. Moreover, cell-type differences have been observed in the IRF3 pathway modulating IKKγ/NEMO alternative splice product [10]. For these reasons, we will focus on the epithelium, the primary sentinel cell of respiratory RNA virus interactions.

Cross-talk between the NF-κB and IRF3 signaling arms is critical for determining the cellular outcome of viral infection. Studies in NF-κB - deficient cells have shown that the initial kinetics of the type I interferon (IFN) response depends on concurrent NF-κB activation [14]. In the absence of NF-κB, the rapid response of IFNβ expression is blunted, reducing the propagation of anti-viral signals in the mucosal surface. Moreover, NF-κB controls expression of the downstream IFN auto-amplification loop through STAT1, IRF-1, 5, and -7 transcription factors. These findings indicate that the two NF-κB and IRF3 signaling arms are highly interconnected and that these interconnections influence the kinetics of the IIR.

The specific purpose of this study is to apply an estimation-validation modeling approach to investigate IIR network topology and interaction rates are consistent with the observed responses of both pathways of human A549 airway epithelial cells (hAECs) in response to dsRNA stimulation, which is a synchronized model of viral infection. dsRNA is a molecular pattern that activates both TLR3 and RIG-I-MAVS signaling in airway epithelial cells and is chosen as a model for viral infection for two reasons. First, it mimics the viral intermediate-products sensed by the innate immune-response mechanism without confounding effects of IIR antagonism produced by the viral proteins, and second, the dsRNA stimulation is synchronous for all cells in the population. Our strategy for determining this topology is composed of two elements: (i) measurements of the time series of major mRNA and protein species in responses to various doses of dsRNA, and (ii) knocking down expression of selected nodes of the pathway, followed by analysis of the responses of the perturbed system. The time courses of mRNA and protein expression of unperturbed and perturbed systems are measured by quantitative real time PCR (Q-RT- PCR) and stable isotope dilution-selective reaction monitoring (SID-SRM) for fitting the model parameters. Quantitative SID-SRM measurements are much more accurate than other conventional blotting methods; this represents the first example to our knowledge of using SRMs for modeling signaling systems. For the siRNA silencing experiments, essential for validation of the model, hAECs were transfected with siRNAs specifically targeting the Pattern Recognition Receptors (PRR) (TLR3, TRIF and RIG-I), the regulatory subunit of IκB kinase complex (IKKγ), and components of the NF-κB pathway (IKKα/β, RelA) and IRF3 pathway (TBKι, IRF3). Our model represents the observed phenomena of dsRNA-inducible RelA and IRF3 activation, negative NF-κB -IRF3 cross-talk, distinct phases of inducible RIG-I degradation and resynthesis. The presence of negative cross-talk is independently confirmed by evolutionary footprint analysis, where we can identify candidate binding sites for NF-κB on IRF genes and vice-versa. The experimentally refined model also predicts the existence of an IRF3-independent mode of RIG-I regulation. In validation experiments, we demonstrate that this transcription factor is IRF7. Finally, we visualized NF-κB and IRF3 translocation from cytoplasm to the nucleus in individual cells using dynamic imaging. The essence of our approach is to use mathematical modeling not only to reproduce the deterministic cell-population data, but also the stochastic single-cell data and reconcile one with the other. This approach has never been used for systems of such complexity.

## Methods

### Experimental Treatments

#### Ethics Statement

Animal manipulations were performed in the UTMB Animal Resource Center (ARC) under the direct supervision of the UTMB Animal Care and Use Committee (ACUC, assurance number A3314-01). The Animal Resource Center is AAALAC (Association for the Assessment and Accreditation of Laboratory Animal Care International) accredited. The ARC follows all standards for AAALAC and international IACUC (Institutional Animal Care and Use Committee) compliance and is staffed by full-time veterinarians and staff overseeing all program aspects. MEF isolation has been approved by the UTMB IACUC under animal protocol 0105020B.

#### Electroporation of dsRNA

Double-stranded RNA (dsRNA) was synthesized using T7 RNA polymerase on a 400 bp luciferase template in pCRII (InVitrogen) with flanking T7 RNA promoters and purified according to manufacturer's recommendations (Ambion). 4 µg of dsRNA was electroporated into hAECs cells at about 5×10^5^ dsRNA units per cell. To ensure experimental reproducibility, in later experiments of dose-response, synthetic dsRNA (polyinosinic–polycytidylic acid sodium salt [poly (I:C)], Sigma [St. Louis, MO]) was substituted for enzymatically synthesized dsRNA. The treated cells were harvested at 18 hr following electroporation and the total RNA of the cells was extracted for further measurements.

#### Knock-down experiments

Experiments were carried out using target gene-specific siRNA and the control nonspecific siRNA, which were reverse- transfected into hAECs cells at the concentration of 100 nM siRNA using TransIT-siQUEST transfection reagent (Mirus Bio Corp) as described previously [15]. Sources of the siRNAs were obtained from Thermo Scientific, Dharmacon, Pittsburgh PA; siRNAs targets: RELA (acc. no.: NM_021975, cat. no.: L-003533-00-0005), IRF3 (acc. no.: NM_001571, cat.no.: L-006875-00-0005), RIG-I (acc. no.: NM_014314, cat. no.: L-012511-00-0005), IKKγ (acc. no.: NM_003639, cat. no.: L-003767-00-0005), Control siRNA (cat. no.: D-001810-10-05). In the present study, the transfection of siRNA was performed 66 hr before dsRNA electroporation, i.e., 72 hr before harvesting the cells.

IRF3/7^−/−^ double knockout mouse embryonic fibroblasts (MEFs [16];) were obtained as a gift from Slobodan Paessler (UTMB). MEFs were established as explant cultures from 14 d old embryonic cultures.

#### Quantitative real time PCR (Q-RT-PCR)

The methods of gene expression analyses using Q-RT-PCR were as described previously [15]. Briefly, 1 µg of RNA was reverse-transcribed using Super Script III in a 20 µl reaction mixture. One µl of cDNA product was amplified in a 20 µL reaction mixture containing 10 µL of SYBR Green Supermix (Bio-Rad) and 0.4 µM each of forward and reverse gene-specific primers. The reaction mixtures were aliquoted into Bio-Rad 96-well clear PCR plate and the plate was sealed by Bio-Rad Microseal B film before putting into PCR machine. The plates were denatured for 90 s at 95°C and then subjected to 40 cycles of 15 s at 94°C, 60 s at 60°C, and 1 min at 72°C in an iCycler (BioRad). PCR products were subjected to melting curve analysis to assure that a single amplification product was produced.

Quantification of relative changes in gene expression was calculated using the ΔΔCt method [17]. In brief, the ΔCt value was calculated (normalized to GAPDH) for each sample: ΔCt [Ct (target gene) − Ct (GAPDH)]. Next, the ΔΔCt was calculated as: (ΔCt (experimental sample) −ΔCt (control sample)). Finally, the fold differences between experimental sample and control sample were calculated using the formula 2^−ΔΔCt^. The results were represented as the normalized fold change compared with control cells transfected with scrambled (non-target control) siRNA. Quantification of absolute concentrations of the short and spliced RNA transcripts was performed by estimating transcript number relative to serial dilutions of cDNA standards in RT-PCR. mRNA expression of RelA, IRF3, RIG-I, IKKγ, TNFAIP3, IκBα, IL8, and IL6, IFNβ and RANTES were detected using gene specific primers [10]. ISG56, ISG54, CIG5, and ISG60 mRNA were measured using gene specific primers (Table 1).

**Table 1:** Sequences of qRT-PCR primers for human IRF3 dependent genes.

	Forward Primer	Reverse Primer
ISG56	5′-TCAGGTCAAGGATAGTCTGGAG-3′	5′-AGGTTGTGTATTCCCACACTGTA-3′
ISG54	5′-GGAGGGAGAAAACTCCTTGGA-3′	5′-GGCCAGTAGGTTGCACATTGT-3′
CIG5	5′-TGGGTGCTTACACCTGCTG-3	5′-GAAGTGATAGTTGACGCTGGTT-3′
ISG60	5′-AAAAGCCCAACAACCCAGAAT-3′	5′-CGTATTGGTTATCAGGACTCAGC-3′

NF-κB dependent gene expression at different dosages of dsRNA was performed by the following procedure: hAECs cells grown in10 cm dishes were trypsinized and electroporated at dsRNA concentrations of 0, 0.1, 1, 5, 10, and 20 µg respectively. The electroporated cells were harvested 18 hr later, and total RNA was extracted for quantification of TNFAIP3, IκBα, IL8, IL6, Groβ and RANTES gene expression by Q-RT-PCR.

#### Cytoplasmic (CE) and nuclear extracts (NE)

hAECs cells were scraped and subjected to hypotonic buffer/detergent lysis [18]. The supernatant (CE) was saved and the nuclear extract (NE) was purified by centrifugation through a sucrose cushion followed by extraction in Buffer C (50 mM HEPES, pH 7.9, 10% glycerol, 400 mM KCl, 1 mM EDTA, 1 mM EGTA, 1 mM DTT, 0.1 mM PMSF) with protease inhibitor cocktail (Sigma Aldrich, St. Louis, MO). Protein content was estimated by Coomassie Brilliant Blue staining using BSA as a standard (Bio-Rad, Hercules, CA).

#### Stable isotope dilution (SID) - Selected Reaction Monitoring (SRM) assays

Protein extracts were denatured, reduced and alkylated with 30 mM of iodoacetamide for 2 hr at 37°C as previously described [19]. The samples were diluted 10-fold with 100 mM ammonium bicarbonate, and digested with 2 µg of trypsin overnight at 37°C. The tryptic digests were dried and resuspended in 5% formic acid-0.01% TFA prior to analysis.

Stable isotope standard (SIS) peptide stocks were diluted to a concentration of 10 fmol/µL with 0.01% TFA. Before LC-SRM-MS analysis, 30 µL of each tryptic digest was mixed with 10 µL of each SIS peptide. These sample solutions were desalted with Waters Sep-Pak C18 cartridge (Milford, MA) prior to SRM analysis.

Selected Reaction Monitoring (SRM) assays were performed as described [19]–[21]. For each high-responding signature peptide, 3–5 y-ions were selected for measurement using optimized collision energy (CE) for each signature peptide. LC-SRM-MS analysis was performed with a TSQ Vantage triple quadrupole mass spectrometer equipped with nanospray source (ThermoFinnigan, San Jose, CA). The online desalting and chromatography were performed using an Eksigent NanoLC-2D HPLC system (AB SCIEX, Dublin, CA). An aliquot of 10 µl of each of tryptic digests were injected on a C18 peptide trap (Agilent, Santa Clara, CA), desalted with 0.1% formic acid at a flow rate of 2 µL/min for 45 min. Peptides were eluted and separated on a reverse-phase nano-HPLC column (PicoFrit, 75 µm×10 cm; tip ID 15 µm) at a flow rate of 500 nL/min with a 20-min linear gradient from 2–40% mobile phase B (0.1% formic acid-90% acetonitrile) in mobile phase A (0.1% formic acid). The TSQ Vantage was operated in high-resolution SRM mode with Q1 and Q3 set to 0.2 and 0.7-Da Full Width Half Maximum (FWHM). All acquisition methods used the following parameters: 1800 V ion spray voltage, a 275°C ion transferring tube temperature, a collision-activated dissociation pressure at 1.5 mTorr, and the S-lens voltage used the values in S-lens table generated during MS calibration. All SRM data were processed using Xcalibur 2.1 using default values for noise percentage and base-line subtraction window, and manually inspected.

#### Dynamic live cell imaging of EGFP-RelA and Strawberry IRF3 transfected hAECs

EGFP-RelA and Strawberry-IRF3 stable hAECs were split into a 6 well culture plate containing collagen-coated 25 mm round cover slips. The cells were then electroporated *in situ* with different dosages of poly IC. Dynamic live-cell imaging was performed by confocal microscopy [11] using a Nikon TiE inverted microscope fitted with a Prairie Technologies Inc. swept field confocal scanhead and an automated focus stabilization system (Perfect Focus, Nikon). After electroporation, the cover slips were immediately placed into a chamber where cells were stably maintained at 37°C with humidified 5% CO_2_. Samples were excited using 488-nm and 561-nm laser lines for EGFP and mStrawberry respectively. Images were captured using a high numerical aperture oil immersion lens (Nikon Super Fluor 40× 1.3NA oil) and a high sensitivity EMCCD camera (QuantEM, Photometrics). The time lapse images for each sample were acquired at 6-min intervals using a multilocation time series protocol controlled by Prairie View software..

### Evolutionary TF Footprint Analysis

Evolutionary conservation of TFBS was performed as described in Iwanaszko et al. [22]. Promoter sequences were obtained using UCSC Genome Browser [23] and were analyzed using NucleoSeq [24] and Consite [25], in search for IRF and NF-κB family TFBSs. Motifs for the NF-κB family, as well as for IRF1 and IRF2 were obtained from the Jaspar [26], while the motifs for IRF3 and IRF7 were obtained from Lin et al. [27].

### Model Building and Analysis

Because our model building is iterative and tightly intertwined with experimental results, it seems more appropriate to defer details to the Results section. Here, we limit ourselves to essentials. Briefly, the work on model building proceeded in the following phases.

Phase 1: Determination of model network topology and couplings

Phase 2: Estimation of parameters based on time series data

Phase 3: Validation of the model based on the knockdown and knockout data

Phase 4: Examination of extrinsic and intrinsic stochasticity and dose-dependence.

#### Simulations

The model was simulated using the bioPN software package, based on the Petri Nets formalism [28], available at http://www.stat.rice.edu/~mathbio/bioPN/. Briefly, bioPN may compute deterministic trajectories of chemical reaction systems using systems of ordinary differential equations, as well as stochastic trajectories in systems with finite number of molecules, using the Gillespie algorithm and its refinements. System of ordinary differential equations and pseudocode corresponding to the bioPN code can be found in Table ST1 and ST3 in File S1.

#### Nuclear translocation and periodogram analysis

Quantification of nuclear translocation of endogenous fluorescent protein was performed on individual cells using CellTracker software [29]. Periodogram analysis was used to identify periodic behavior of nuclear translocation. This is a version of spectral analysis, which allows estimating the relative weights of components with different periodicities in the observed dynamics. The R-function “prdgrm” used to compute periodograms was implemented in bioPN ([28], available at http://www.stat.rice.edu/~mathbio/bioPN/).

## Results

To understand the interactions between the two arms of the IIR signaling pathway, we probe the topology of couplings and dynamic coordination using time series measurements of key mRNA expression and protein abundance measurements, as well as in response to siRNA knockdowns of major regulatory points of the pathway. We use an estimation-validation approach to devise a mathematical model of the epithelial IIR; this model exhibits a cell-type dependent structure determined based on literature data and parameter values determined based on its fit to time series data and validated by knockdown data. To accomplish this end, we first provide key experimental findings that inform the model topology and parameters, followed by mathematical model building and validation.

### Experimental Findings on Model Topology and Kinetics

#### Kinetics of NF-κB and IRF3 dependent gene expression

**Figure 1:**
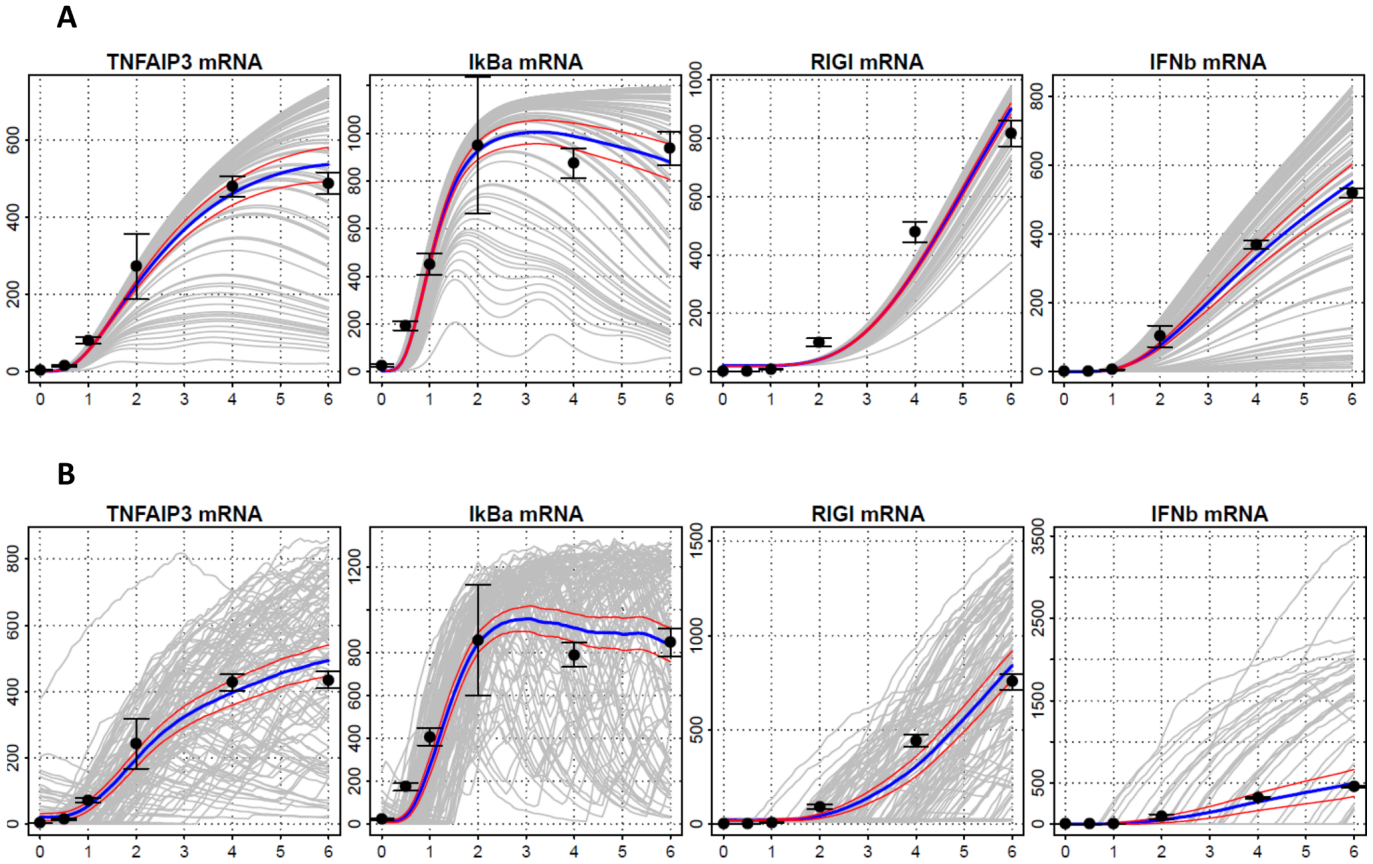
Model Estimation. Time series of mRNA levels of TNFAIP3, IκBα, RIG-I and IFNβ following stimulation by 4 µg dsRNA in hAECs cells for 0, 0.5, 1, 2, 4, and 6 hr. Gene expression estimated using Q-RT-PCR was as described previously [10]. Experimental measurements, black circles with empirical 95% confidence intervals based on triplicate measurements; means of 100 simulated single-cell trajectories, blue lines; 95% confidence bands based on simulations, red lines. Two types of simulations presented (A) under extrinsic noise, (B) under extrinsic and intrinsic noise. Horizontal axis - time (hr); vertical axis - number of molecules. Absolute values of experimental measurements scaled to simulation data (see the text for details).

Previously, we demonstrated that RNA virus infection activates both the TLR3 and RIG-I signaling pathways in human hAECs as a model airway epithelial cell [13], [15], [30]. To understand the kinetics of pathway activation, hAECs were synchronously stimulated with intracellular dsRNA (4 µg) by electroporation for a time series of 0, 0.5, 1, 2, 4, and 6 hr. Total cellular RNA was extracted and subjected to Q-RT-PCR for determination of relative changes in NF-κB and IRF3 dependent genes (Fig. 1, black solid circles). Data were scaled in a way explained further in the paper. These data indicated that dsRNA strongly induced the expression of the *TNFAIP3/A20* and *NFKBIA/IκBα* genes; we noted that these selectively NF-κB dependent genes were the most rapidly induced, whose expression could be detected within 30 min of stimulation. In contrast, the expression of type I IFN (IFNβ) was more delayed, and was not detectably changed until 2 hr after dsRNA stimulation. To be discussed later, we also noted that RIG-I mRNA induction had a similar kinetic response as that of IFNβ. All of this together, these data indicate that the NF-κB dependent genes are most rapidly inducible in response to dsRNA.

#### Signal inducible RIG-I degradation and synthesis

**Figure 2:**
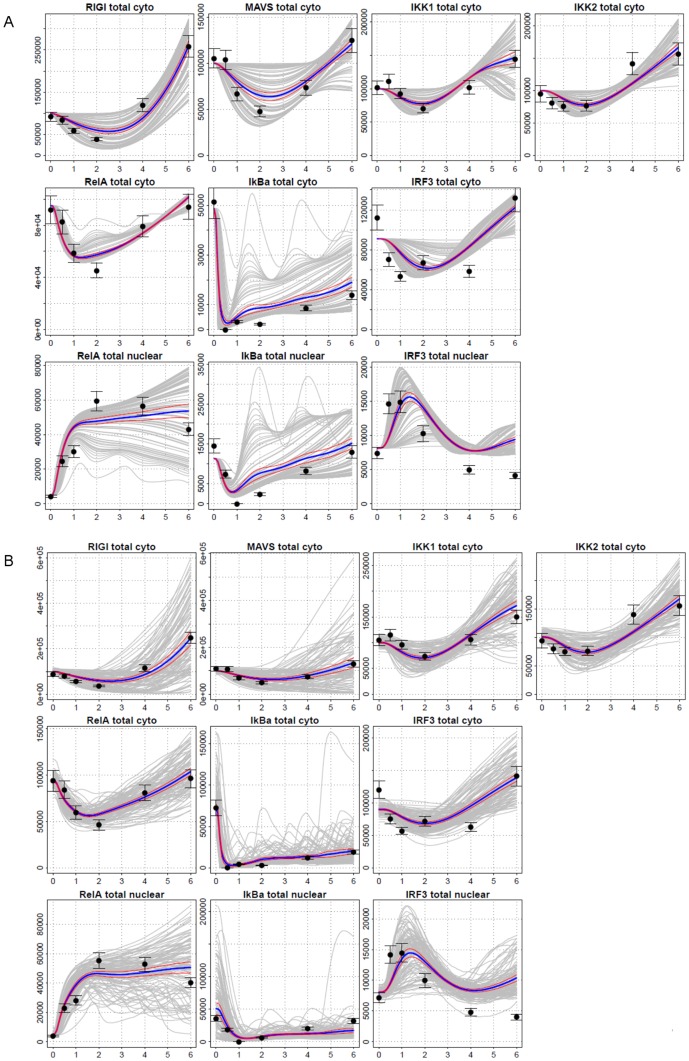
Model Estimation. Time series of phosphorylated proteins, following stimulation by 4 µg dsRNA in hAECs, obtained using the Selected Reaction Monitoring (SRM) assay: In the cytoplasm, RIG-I, MAVS, IKK1, IKK2, RelA, IκBα, and IRF3; and in the nucleus, RelA, IκBα, and IRF3. Experimental measurements, black circles with empirical 95% confidence intervals based on triplicate measurements; means of 100 simulated single-cell trajectories, blue lines; 95% confidence bands based on simulations, red lines. Two types of simulations presented (A) under extrinsic noise, (B) under extrinsic and intrinsic noise. Horizontal axis: time (hr); vertical axis: number of molecules. Absolute values of experimental measurements scaled to simulation data (see the text for details).

The SID-SRM assays are quantitative assays to determine the abundance of target proteins in subcellular fractions [20]. A time course of dsRNA-stimulated hAECs cells was fractionated into cytoplasmic preparations and subjected to SID-SRM analysis for RIG-I, MAVS, IKK1 and IKK2 (Fig. 2, top panels). Each of these cytoplasmic proteins exhibited complex behavior, with RIG-I and MAVS being markedly reduced to less than half their initial abundances within 2 h after dsRNA exposure before they are inducibly resynthesized to levels greater than that of control (Fig. 2, top panel, black solid circles). IKK1 and IKK2 showed similar depletions, although the magnitude was significantly less. Interestingly, the parallel depletion of both RIG-I and MAVS are consistent with their known regulation by the RNF E3 ubiquitin ligase [31]. Although we cannot exclude a small component of translational inhibition, the dramatic later phase accumulation of RIG-I protein (Fig. 2), interpreted together with our earlier mRNA analysis (Fig. 1) that RIG-I mRNA is dramatically upregulated indicate that the expression of RIG-I protein is strongly induced by dsRNA exposure. By comparison, it is well established that NFKBIA/IκBα is a negative regulator of the NF-κB/RelA pathway whose abundance is regulated by inducible phosphorylation-ubiquitylation [32]. A similar pattern of inducible degradation and resynthesis of IκBα is also observed. Jointly, these data indicate that RIG-I is under coordinated control by signal-induced transcription and proteolytic degradation.

#### Kinetics of NF-κB and IRF3 activation and nuclear translocation

For the proteins known to undergo nuclear translocation, the cytoplasmic and nuclear fractions were measured for RelA, NFKBIA/IκBα and IRF3 abundance (Fig. 2, bottom panels). From this analysis, we noted that the cytoplasmic fraction of cytoplasmic NF-κB/RelA is initially depleted from the cytoplasm by 0.5 hr, where its nuclear abundance increases. We note that the nuclear abundance of NF-κB/RelA does not reach steady state until 2–4 hr after dsRNA transfection (Fig. 2, black solid circles). By contrast, the cytoplasmic fraction of IRF3 is somewhat faster and transiently depleted, detectable within 0.5 hr and peaking after 1 hr of stimulation, followed by its cytoplasmic reaccumulation to above pre-treatment levels at 6 hr (Fig. 2, bottom right panel, black solid circles). These observations are consistent with those of others that observed viral-induced IRF3 turnover and resynthesis in epithelial cells [33] and of our separate studies quantifying IRF3 in SID-SRM experiments [20]. Cytoplasmic IRF3 depletion initially correlates with its transient translocation into the nucleus from 30 min to 1 hr, and returning to low levels thereafter (Fig. 2, black solid circles). Together, these data indicate that the activation profiles of IRF3 and NF-κB are distinct; with activation of IRF3 being transient and slightly preceding that of NF-κB (see Fig. 2).

#### Cross-inhibition effect between IRF3 and NF-κB pathways

**Figure 3:**
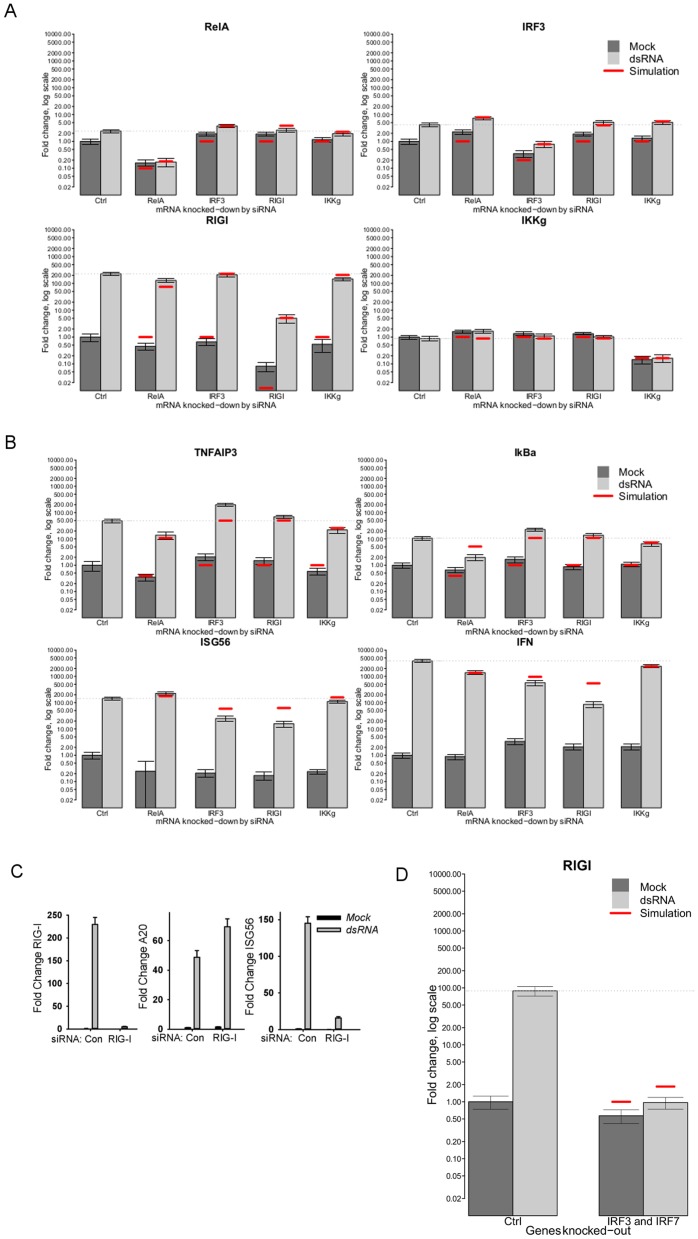
Model Validation. Gene knockdowns using siRNA specific to target genes in hAECs. The experiments were carried out using target gene-specific siRNA and the control nonspecific siRNA, which were reverse-transfected into hAECs at the concentration of 100 nM. The data presented are the corresponding mRNA levels at 6 hr after electroporation (fold change, in logarithmic scale). (A), knockdown of RelA, IRF3, RIG-I and IKKγ. (B), knockdown of TNFAIP3/A20, NFKBIA/IκBα, ISG56 and IFNβ. The mRNA levels of the indicated genes (at top) were determined by RT-PCR. Results were represented as normalized fold change of expression compared to control non dsRNA-induced cells transfected with scrambled (non-target control) siRNA. Gray bars, experiment (dark, no dsRNA stimulation; light, 4 µg dsRNA); red lines, model simulation. 95% confidence intervals of experimental data (based on 3 replicates) are distorted by the logarithmic scale. In linear scale, the relative error rate is approximately 10%. Please notice that in IFNβ and ISG56 charts, the model values at time 0 are equal to 0, which is impossible to depict on the log scale. (C), Effect of RIG-I knockdown on NF-κB and IRF3 dependent gene expression. hAECs were transfected with siRNA to RIG-I or control siRNA (Con). Left panel, effect of RIG-I siRNA on RIG-I expression. Note that dsRNA induced RIG-I expression is largely inhibited in hAECs transfected with RIG-I siRNA. Middle panel, effect of RIG-I knockdown on NF-κB-dependent TNFAIP3/A20 gene expression. dsRNA induces TNFAIP3/A20 expression in RIG-I knockdown cells. Right panel, effect of RIG-I knockdown on IRF3-dependent gene expression. RIG-I knockdown significantly blunts IRF3-dependent ISG56 expression. We conclude from these data that RIG-I is primarily coupled to IRF3 signaling in hAECs. (D), Effect on RIG-I expression in murine MEF cells with both IRF3 and IRF7 genes knocked down. RIG-I is down-regulated, which suggests IRF7 may play a key role in RIG-I up-regulation. Compare with RIG-I results in Panel A where the IRF3 knock-down does not seem to down-regulate RIG-I expression.

Our data indicates that dsRNA activates temporally distinct gene expression patterns and transcription factor translocation in hAECs (Fig. 1 and 2). To identify downstream targets of the IRF3 and NF-κB transcription factors, we explored the effect of siRNA mediated silencing. For this purpose, hAECs cells were transfected with duplex target (or scrambled) siRNAs specific to the NF-κB pathway (IKKγ and RelA) or to IRF3 pathway (RIG-I and IRF3) prior to intracellular dsRNA stimulation. The extent of siRNA knockdown of target genes was evaluated by Q-RT-PCR. As seen in Fig. 3, mRNA levels of RelA, IRF3, RIGI and IKKγ were measured in control and dsRNA-transfected cells. Gene knockdown experimental results are presented as dark gray bars (without dsRNA) and light gray bars (4 µg dsRNA). Note that the fold change in mRNA expression is depicted on a log-scale.

Examination of these experiments showed that dsRNA transfection induced RelA expression in control siRNA-transfected cells by 2-fold (Fig. 3A, top left panel). We also were able to reduce RelA mRNA expression to 20% of that produced by control siRNA after RelA siRNA transfection in both unstimulated and dsRNA transfected cells. We further noted that the basal and dsRNA induced levels of RelA were increased by either IRF3 – and RIG-I siRNA silencing, suggesting that RIG-I-IRF3 pathway negatively regulates RelA expression (Fig. 3A, top left panel). In this experiment, both basal and dsRNA induced IRF3 levels were significantly reduced by siRNA to 35% of that in scrambled siRNA control (Fig. 3, top right panel).

We have also observed that mRNA levels of IRF3 were upregulated 3–4 fold in response to dsRNA transfection in control siRNA-transfected cells (Fig. 3A, top right), consistent with its apparent resynthesis (Fig. 1) and reproducing our recent study [34]. Similarly, IRF3 expression was also induced by RelA knockdown in control and dsRNA transfected cells (Fig. 3A, top right panel). These data indicated to us that RelA also negatively regulates IRF3 expression.

The dsRNA induction of TNFAIP3/A20, NFKBIA/IκBα, IL-8 and IL6 expression (IL6 and IL8 results not shown) were also significantly reduced by RelA and IKKγ silencing, indicating that these genes are primarily RelA-dependent (Fig. 3B). We also noted that RIG-I and IRF3 silencing did not inhibit, but rather increased TNFAIP3/A20 and NFKBIA/IκBα expression (Fig. 3B). These data indicated to us that RIG-I is primarily coupled to the IRF3 pathway and that the IRF3-RelA negative cross-regulation is functionally significant.

To experimentally validate the coupling of RIG-I with IRF3 and not NF-αB in hAECs, we examined the effect of RIG-I silencing on NF-κB- and IRF3-dependent gene expression. Transfection of RIG-I siRNA significantly reduced basal and dsRNA induced RIG-I expression (Fig. 3C). Although RIG-I silencing produced a greater induction of TNFAIP3/A20 in response to dsRNA, the expression of ISG56, a IRF3-dependent gene, was significantly inhibited (Fig. 3C).

**Figure 4:**
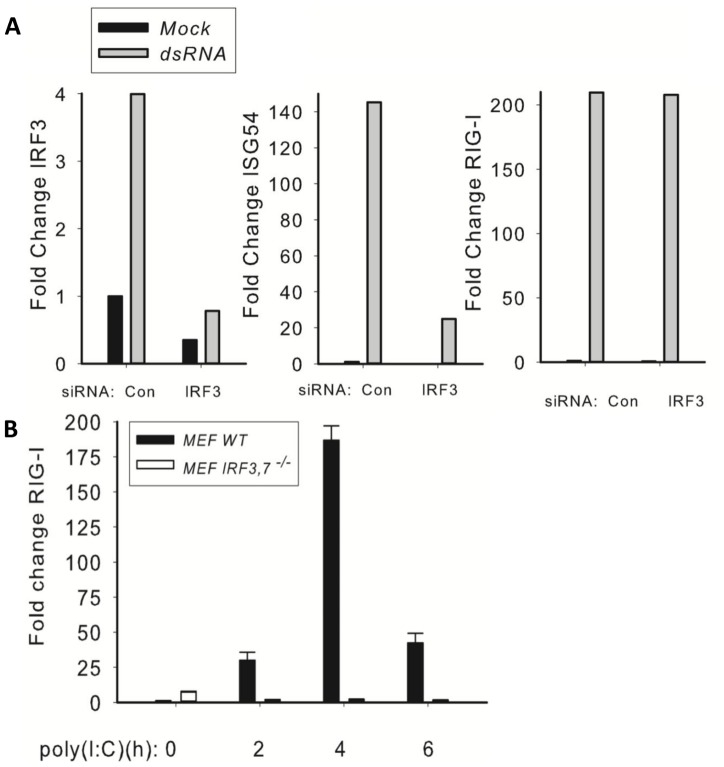
Model Validation. RIG-I synthesis is IRF7 dependent. (A) IRF3 siRNA knockdown of A549 cells. A549 cells were transfected with scrambled control (Con) or IRF3-specific siRNAs and stimulated in the absence or presence of poly(I:C). mRNA measured by Q-RT-PCR and the results are represented as the normalized fold change expression compared to control cells transfected with scrambled (non-target control) siRNA. Shown are normalized mRNA expression for IRF3, RIG-I and ISG56 mRNA on a linear scale. Note that poly(I:C) induced ISG56 expression is reduced by 86%, whereas RIG-I expression is reduced by <10%. (B) Effect of IRF3/IRF7 deficiency on RIG-I expression. WT or IRF3/IRF7-/- MEFs were transfected with poly(I:C) and expression of RIG-I determined by Q-RT-PCR. Shown is a time course of wild type or IRF3/7-/- double knockout cells in response to dsRNA. Note that RIG-I expression is completely blocked in IRF3/7-/- cells. Together these data indicate that RIG-I expression is largely independent of IRF3, but requires IRF7.

RIG-I mRNA expression was also induced by dsRNA treatment, and was not affected by IRF3 knockdown (Fig. 3 and 4). The observation that IRF3 knockdown has no effect on RIG-I expression is significant because previous studies from another group has suggested that RIG-I expression is dependent on downstream IRF3-IFN signaling [35].

#### Bioinformatics evidence for interaction between the IRF3 and NF-κB pathways

Using computational methods and cross-species comparisons between human, chimpanzee, mouse and cattle, we analyzed promoters (1 kb upstream of transcription start sites) of genes for IRF3 and NF-κB TFs. Using a similar method of TFBS analysis described in Iwanaszko et al. [22], we identified TF binding sites (TFBSs) across an index promoter region and then analyzed if these TFBSs were conserved among species in homologous domains. Analysis of TFBSs in the IRF gene family indicate that the IRF genes have a greater enrichment of NF-κB binding sites than that of IRF TFBS, suggesting that NF-κB may modulate IRF family expression (Table ST4 in File S1). We have found that the IRF3 binding motif is not widely represented in our dataset, but the location of binding sites that were found, may be crucial in the view of other TFBS. We have also found binding motifs for the NF-κB family, mostly for the transcriptionally active Rel A subunit, in the IRF7 gene (Table ST4 in File S1). This evolutionary conservation matches the experimental validation of IRF7 expression under NF-κB control [36]. As for the activity of IRF7, we have found that IRF7 binding sites are present in nearly all genes coding for transcription factors in our dataset, with the exception of NFKB2 and a few IRF1 promoter variants. IRF7 binding sites are also present in genes important in higher level control of the IRF and NF-κB arms, including genes such as MAVS, RIG-I, IKK1 and IKK2. IRF7 binding sites have been found in both human and murine promoters, but we note that the conservation of motifs differs among analyzed genes. A detailed analysis has been submitted as a separate publication. In summary, we confirmed an intricate pattern of cross-activation between IRF and NF-κB family, suggested by the knockdown experiments (Fig. 1).

#### IFNβ expression is dependent on both NF-κB and IRF3 signaling in the IIR

The production of type I IFN is the hallmark of the IIR whose function is to limit viral spread until the activation of adaptive immunity [37]. IFNβ activation occurs at the level of gene expression through IRF3-dependent chromatin remodeling event, known as an enhanceosome [38]. Although NF-κB is a component of the enhanceosome, IFNβ is primarily an IRF3 dependent gene. Although IFNβ expression was predictably reduced by RIG-I and IRF3 silencing we found that IFNβ expression was also inhibited by ∼50% by RelA silencing compared with that in control, indicating that NF-κB/RelA is a rate-limiting step for IFNβ expression (Fig. 3B).

#### RIG-I expression response to significant perturbations in IIR signaling

RIG-I expression is highly inducible in response to dsRNA, with ∼220-fold induction of mRNA expression. However, its expression is not affected by IRF3 knockdown and reduced only about 2-fold by RelA knockdown (Fig. 3A). All of these data suggest that RIG-I expression is controlled by some other transcription factors activated in response to virus exposure. To more fully understand the mechanism of RIG-I induction, we next examined dsRNA-induced RIG-I expression in IRF3 knochdown (Fig. 4A) and IRF3/7^−/−^ double knock out (DKO) cells (Fig. 4B). Although RIG-I expression was not affected by IRF3 knockdown, RIG-I expression was significantly inhibited in the DKOs. Examination of the evolutionary footprint analysis of the RIG-I promoter, significant enrichment of IRF7 binding sites is seen (Table ST4 in File S1). Based on these results, we concluded IRF7 is a primary regulator of RIG-I expression.

#### ds-RNA Dose-dependence of transcription and translocation

**Figure 5:**
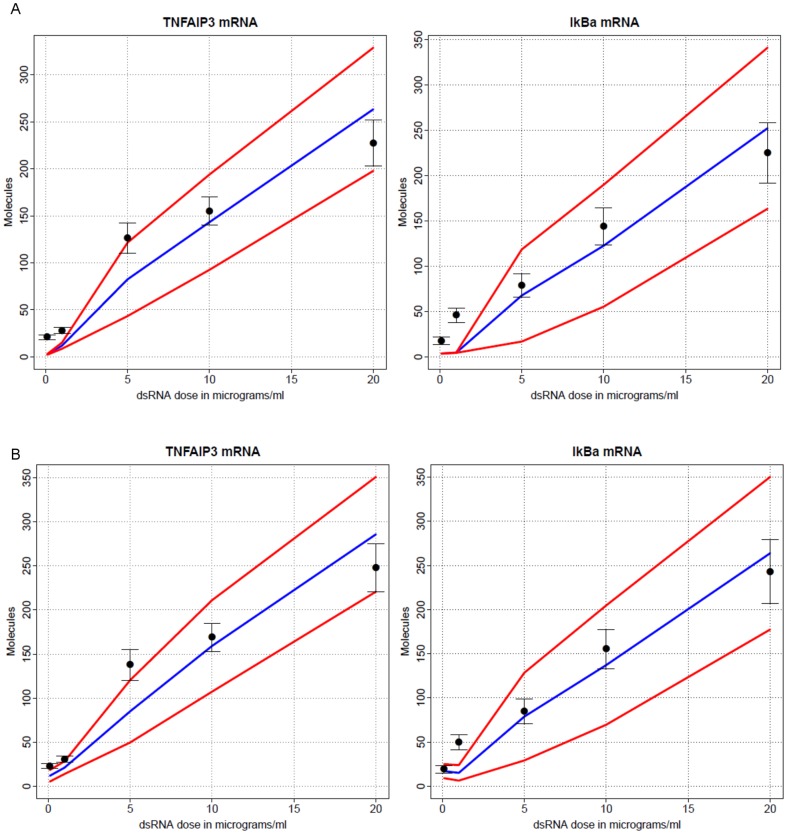
Observed and simulated dsRNA dose-dependence curves in hAECs. Experimental measurements, black circles with empirical 95% confidence intervals based on triplicate measurements; means of 100 simulated single-cell trajectories, blue lines; 95% confidence bands based on simulations, red lines. Two types of simulations presented (A) under extrinsic noise, (B) under extrinsic and intrinsic noise. Horizontal axis: dsRNA dose (µg); vertical axis: number of molecules.

We examined the effects of different dosages of dsRNA on the transcription profiles of the NF-κB dependent gene TNFAIP3/A20, NFKBIA/IκBα, IL8, IL6, Groβ and RANTES at 18 hr after electroporation. Increasing concentrations of dsRNA were introduced into cells, and relative changes in mRNA estimated by Q-RT-PCR (Fig. 5). These data showed that dsRNA could increases the transcription levels of all NF-κB dependent genes over this dose range. For most genes, the highest transcription levels were produced in response to 20 µg of dsRNA, the maximal dosages of dsRNA used. However, the highest transcription levels of IL8 and RANTES were reached at 10 µg of dsRNA and their transcription levels at 20 µg of dsRNA sharply decreased, perhaps due cell toxicity and the unstable nature of their mRNA (not shown).

#### Time series of fluorescence protein tagged- RelA and IRF3 distribution at a single-cell level under different concentrations of dsRNA

**Figure 6:**
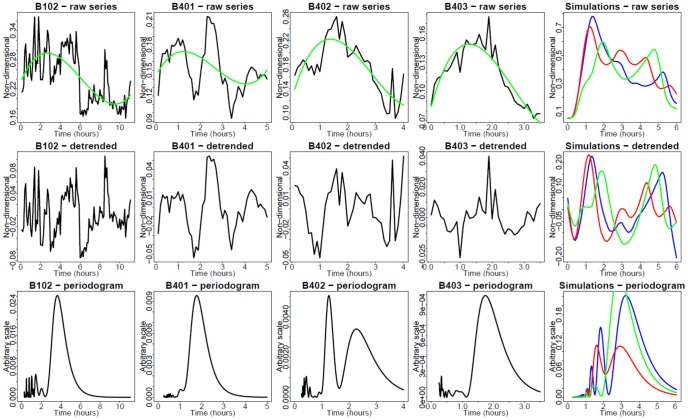
Single cell nucleus/cytoplasm ratios under 5 µg of dsRNA stimulation (RelA, green channel fluorescence). EGFP-RelA stable hAECs were electroporated with different dosages of synthetic dsRNA analog Poly IC and dynamic live cell imaging was performed. Time presented in hr. Green trend lines are third-order polynomials, fitted using least-squares minimization. Upper row: Raw time series. Middle row: Detrended time series. Bottom row: Fourier periodograms. Columns 1–4: Observed single cells. Column 5: Two simulated cells.

**Figure 7:**
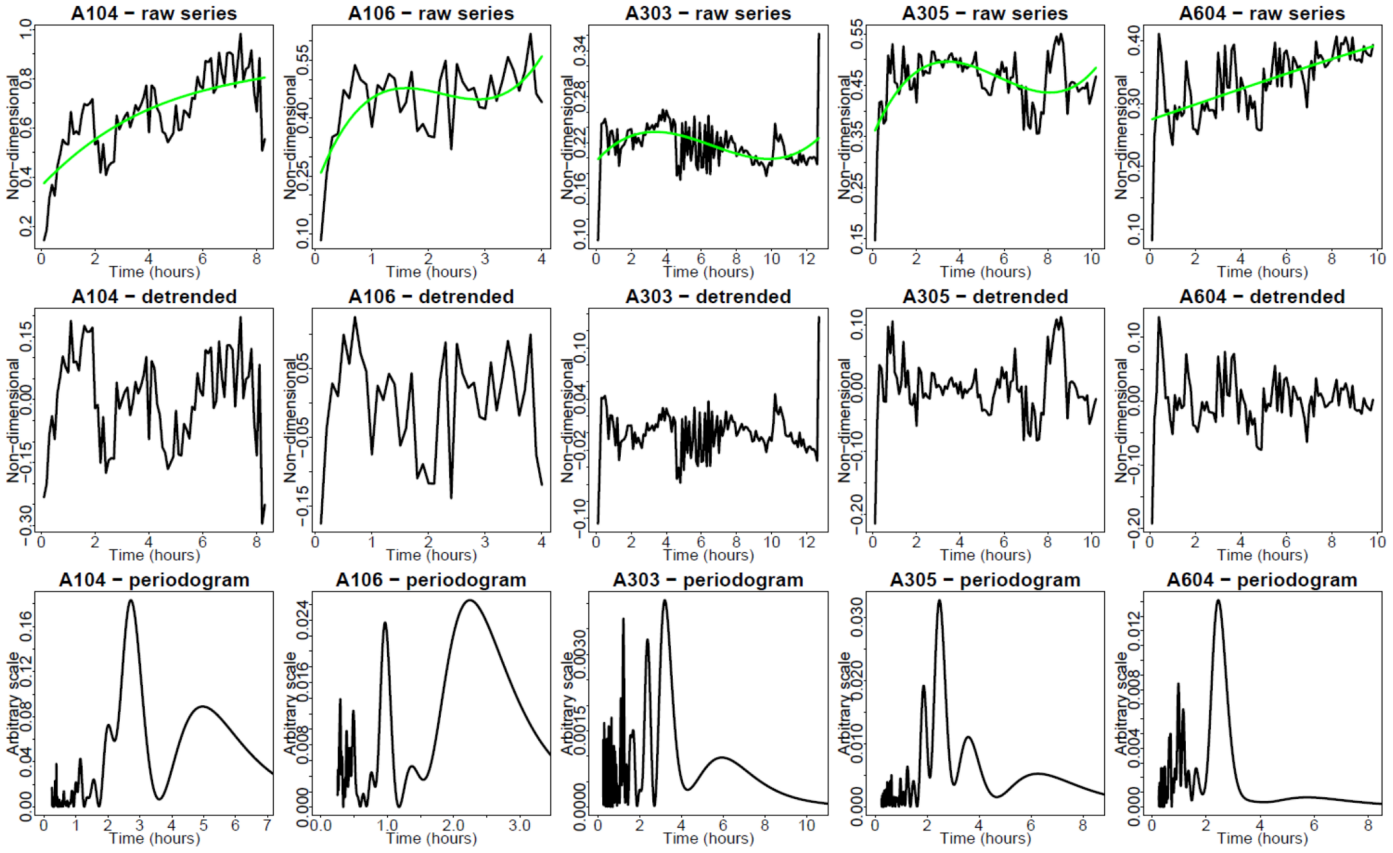
Single cell nucleus/cytoplasm ratios under 50 µg of dsRNA stimulation (RelA, green channel). EGFP RelA stable hAECs were electroporated with different dosages of synthetic dsRNA analog Poly IC and dynamic live cell imaging was performed. Time is in hr. Green trend lines are third-order polynomials, fitted using least-squares minimization. Upper row: Raw time series. Middle row: Detrended time series. Bottom row: Fourier periodograms. Columns 1–5: Observed single cells.

**Figure 8:**
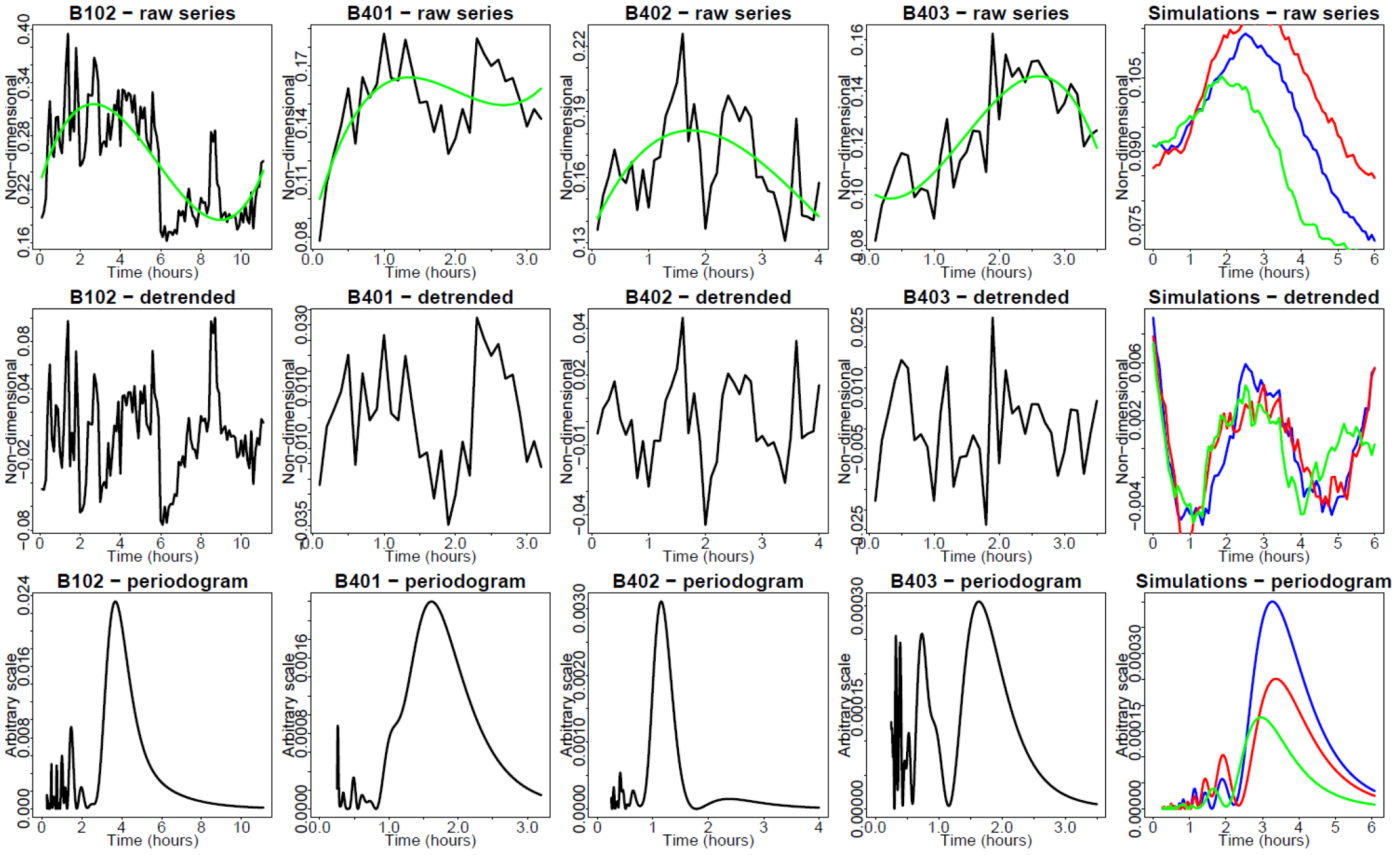
Single cell nucleus/cytoplasm ratios under 5 µg of dsRNA stimulation (IRF3, red channel fluorescence). Strawberry -IRF3 hAECs were electroporated with different dosages of synthetic dsRNA analog Poly IC and dynamic live cell imaging was performed. Time presented in hr. Green trend lines are third-order polynomials, fitted using least-squares minimization. Upper row: Raw time series. Middle row: Detrended time series. Bottom row: Fourier periodograms. Columns 1–4: Observed single cells. Column 5: Two simulated cells.

**Figure 9:**
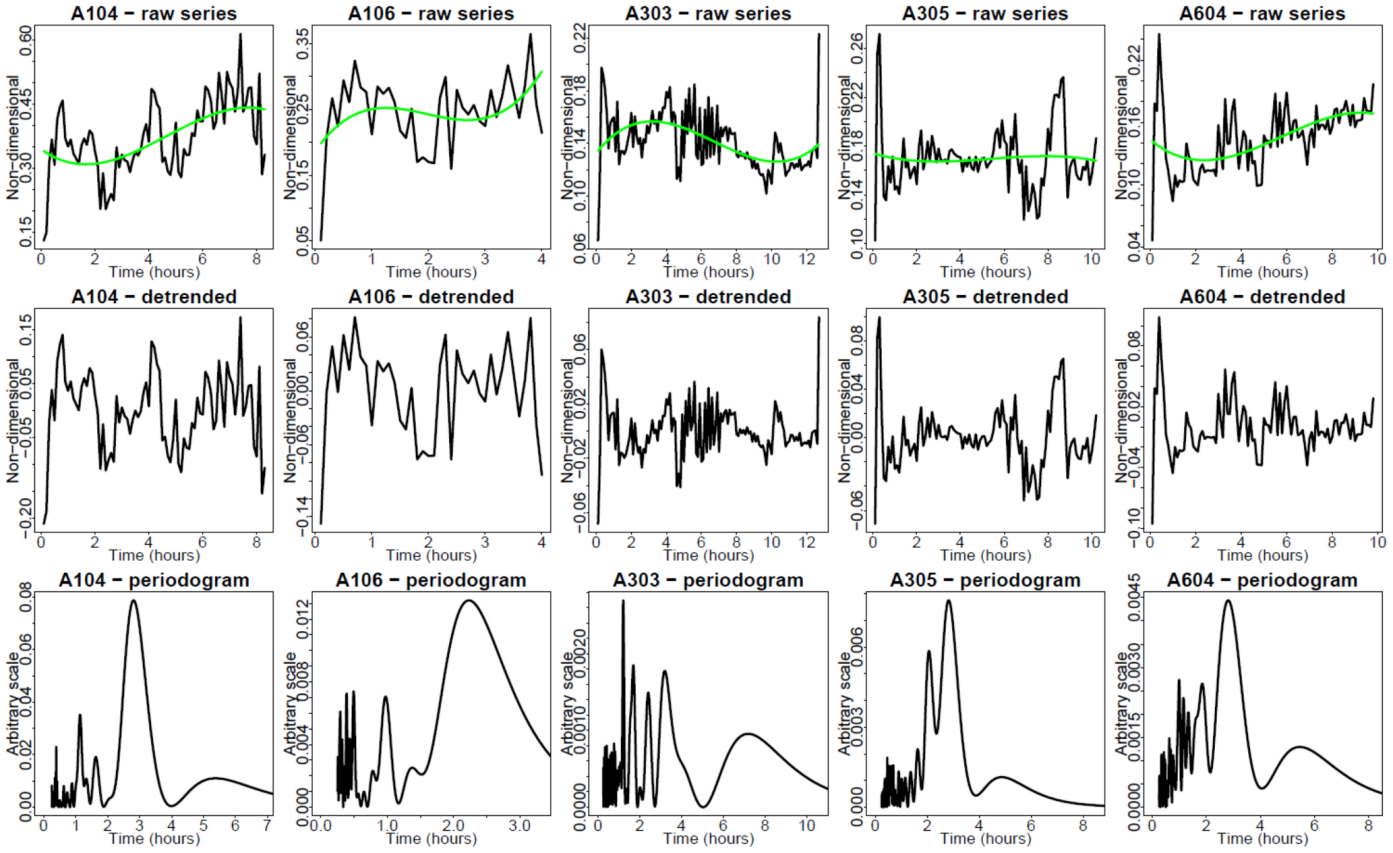
Single cell nucleus/cytoplasm ratios under 50 µg of dsRNA stimulation (IRF3, red channel). Strawberry -IRF3 hAECs were electroporated with different dosages of synthetic dsRNA analog Poly IC and dynamic live cell imaging was performed. Time is in hr. Green trend lines are third-order polynomials, fitted using least-squares minimization. Upper row: Raw time series. Middle row: Detrended time series. Bottom row: Fourier periodograms. Columns 1–5: Observed single cells.

EGFP-RelA and Strawberry-IRF3 stable hAECs were electroporated with different dosages of synthetic dsRNA and dynamic live cell imaging of EGFP RelA and Strawberry IRF3 was performed. We observed that dsRNA could induce both GFP-RelA and Stawberry-IRF3 nuclear translocation. From the imaging data, we could find that the profiles of Poly IC induced GFP-RelA and Stawberry-IRF3 nuclear translocation vary with different dsRNA concentrations. For example, at the level of 5 µg/ml Poly IC (Fig. 6), RelA showed oscillatory nuclear translocation quite different from that in 50 µg Poly IC (Fig. 7). Analogous observation is made concerning the single-cell response of IRF3 (Figs. 8 and 9, respectively).

It should be noted that there is some difference in the time course of RelA and IRF3 translocation in the time series in Figure 2 (cell population data) under 4 ug Poly IC, which is less clear in the 5 ug Poly IC single-cell data.

### Mathematical Model Building and Analysis

Our estimation-validation approach for model building has four distinct phases:

***Phase 1: Determination of model network topology and couplings:-***

**Figure 10:**
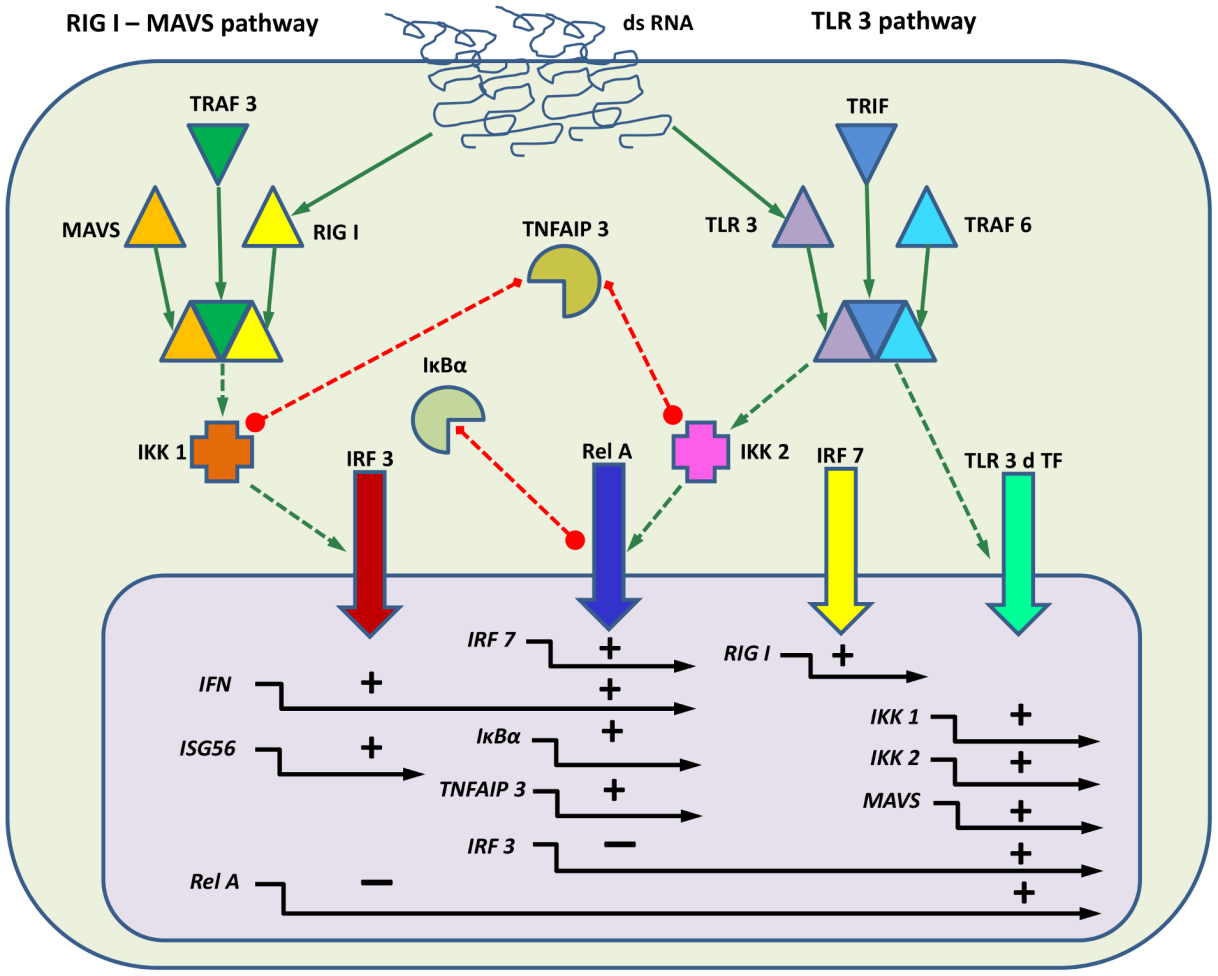
Model Couplings. Simplified schematic of the IRF3-NF-κB model. Only dsRNA, proteins (in the cytoplasm) and genes (in the nucleus) are shown. Solid green lines on the top denote direct chemical binding. Green dotted lines denote activation. Vertical thick colored arrows denote translocation of activated transcription factors into the nucleus. Red dotted lines denote inhibition. Horizontal solid black arrows in the nucleus denote gene transcription, with plus or minus signs denoting activation or repression, respectively. Transcripts and inactive forms of the proteins are omitted for simplicity.

1. Review of literature findings in hAECs was been carried out to propose the topology of the two arms of the IIR (Fig. 10). This process was influenced by feedback from Phase 3 (knockdowns and knockouts), which introduced corrections to the proposed structure. The structure of the couplings was also corroborated by a bioinformatic evolutionary footprint study of TF Binding Sites.

***Phase 2: Estimation of parameters based on time series data:-***

1. Time series (0, 0.5, 1, 2, 4, and 6 hr) of mRNAs of key signaling molecules were measured in order to observe the profiles of changes in NF-κB and IRF3 signaling pathways (Fig. 1).
2. Time series of cytoplasmic and nuclear proteins of key signaling molecules were determined at same time points as above using SID-SRM experiments (Fig. 2)
3. Parameters of the model were estimated based on fit to time series data (Fig. 1,2).

***Phase 3: Validation of the model based on the knockdown and knockout data:-***

1. Knockdowns of genes of interest were performed using small interfering RNA (siRNA) of NF-κB, IRF3, RIG-I, and IKKγ and subsequently stimulated with or without dsRNA (Fig. 3).
2. Model knockdown responses were determined and compared with experimental data (Fig. 3).
3. Experiment using IRF3/IRF7-/- MEFs was performed to address the IRF7-dependence of RIG-I regulation. Results of Phase 3 contributed to refinement of the model structure in Phase 1.

***Phase 4: Examination of extrinsic and intrinsic stochasticity and dose-dependence:-***

1. The dsRNA dose-dependence curve of NF-κB and IRF3 dependent gene expression in hAECs were determined using Q-RT-PCR (Fig. 4).
2. Time series of fluorescence protein-labeled RelA and IRF3 distribution in single-cells under a range of dsRNA concentration were also performed using dynamic confocal microscopy (Fig. 6, 7, 8, 9).
3. Stochastic version of the model was run. Extrinsic stochasticity is due to variability of dsRNA doses absorbed by single cells. Intrinsic stochasticity is mostly due to random initiation and termination of transcription, but a fully stochastic optimized Gillespie algorithm has been run. Oscillations of the model trajectories have been compared to single-cell data (Fig. 1,2,7).
4. Model dose-dependence was determined in both extrinsic alone as well extrinsic and intrinsic stochasticity settings and compared to data (Fig. 5).

It is subject of a debate in the systems biology community whether attempts should be made to build a model based on a “rigorous” method such as Maximum Likelihood Estimation or minimization of a performance index, or using an iterative interactive method as described here. A discussion of literature approaches is presented in the Discussion section.

**Phase 1: Determination of network topology and couplings based on literature data.** ***RIG-I-MAVS and TLR3 molecular pathways following viral infection:*** As RNA virions enter the cells, they lose the envelope and become internalized, which is followed by release of its genome. During the process of transcription/replication, dsRNA is produced [39], and represents the molecular pattern sensed by cytoplasmic RIG-I and endosomal TLR3.

***RIG-I-MAVS pathway:*** Viral by-products sensing: RIG-I/DDX58 is a cytoplasmic RNA helicase that binds dsRNA [40], [41]; RNA-bound RIG-I is rapidly ubiquitinated by E3 ligases (TRIM25 and Riplet/RNF-13) [42]. Signaling complex assembly: RIG-I binds to Mitochondrial anti-viral signaling protein (MAVS; also known as IPS-1, VISA, and CARDIF) [43]; RIG-I-MAVS recruits TNF- Receptor-Associated Factor 3 (TRAF3) [44]. IKK1 activation: RIG-I-MAVS-TRAF3 activates the complex TANK-binding kinase 1 (TBK1), inhibitor of κB kinase ε (IKKε) and IKKγ (called here IKK1) [9], [45]. IRF3 activation: IKK1 phosphorylates IRF3 on COOH terminal serine residues; this process induces dimerization and rapid translocation into the nucleus [46], [47].

***TLR3 pathway:*** Viral by-products sensing: TLR3 is a transmembrane signaling protein that recognizes and binds dsRNA located in the endosomal and extracellular compartments [39]. Signaling complex assembly: TLR3 binds to the adaptor molecule TIR-domain-containing adapter-inducing interferon-β (TRIF) [48]. TLR3-TRIF recruits the TNF- Receptor-associated factor 6 (TRAF6) via a specific TRAF6-binding sequence [49]. IKK2 and TLR3dTF activation: TLR3-TRIF-TRAF6 activates IKKγ-IKKα-IKKβ complex (called here IKK2) [49] and the hypothetical TLR3-dependent transcription factor (TLR3dTF). IκBα degradation, freeing NF-κB: IKK2 phosphorylates IκBα, resulting in its ubiquitylation and proteolytic degradation, freeing NF-κB to enter the nucleus [6], [50].

***“Pool” of NF-κB:*** We hypothesize that not all NF-κB is bound to IκBα. We consider “pool” (non- IκBα-binding fraction) of NF-κB, part of which may be bound to IκBε or other inhibitors. There is a continuous exchange between the “pool” NF-κB and (IκBα-binding) NF-κB. When IKK2 is active, there is a stronger migration from the “pool” NF-κB and NF-κB, which might be interpreted as the part of NF-κB pool bound to IκBε being freed and transferred to the nucleus along with the part bound to the now-degraded IκBα).The existence of the NF-κB reservoir was demonstrated by quantitative dynamic imaging, western immunoblotting, and SID-SRM assays [11], [19].

***Activation of genes by transcription factors:*** Active transcription factors translocate into the nucleus and bind to the cis-regulatory elements in the proximal promoters of target genes. Through the mechanism of protein-protein interactions, transcription factors recruit histone acetyl transferases (p300/CBP) and transcriptional elongation factors (CDK9/Brd4) into an activated state and subsequently induce expression of target genes [15], [51] (Fig. 10).

***Negative feedback loop:*** TNFAIP3/A20 is an NF-κB-dependent gene and a member of the OTU deubiquitinase family. TNFAIP3 could terminate signal pathway activation by Lys63 modifications and thereby inactivate the IKKs. TNFAIP3/A20 removes lysine-63 (K63)-linked ubiquitin chains from receptor interacting protein (RIP), an essential mediator of the proximal TNF receptor 1 (TNFR1) signaling complex [52]. In the model we use the fact that TNFAIP3/A20 binds IKKγ and inactivates (or leads to degradation) of IKK1 and IKK2 (see also ref. [53]).

NFKBIA/IκBα is an NF-κB-dependent gene that binds to free NF-κB and forms an inactive complex with it, and sequesters NF-κB in the cytoplasm [6], [50].

***Mathematical model of the RIG-I-MAVS and TLR3 molecular pathways:*** As stated above, the main sensors of dsRNA are RIG-I and TLR3, and both pathways are capable of activating both IRF3 and NF-κB depending on the variant of virus studied, and/or the cell line used. Our experimental data indicate that RIG-I activates the IRF3 pathway, but not the NF-κB pathway (Fig. 3C). As a consequence, in our current model we consider two interdependent pathways, one for IRF3 regulated by RIG-I, and one for NF-κB, which we assume is regulated by TLR3. It is assumed that NF-κB and IRF3 cross-inhibit their respective transcriptions. Also, TNFAIP3 acts to deactivate IKK1 and IKK2.

Certain complexes have been named to simplify notation: IKKγ-TBK1-IKKε and IKKα-IKKβ-IKKγ are named IKK1 and IKK2, respectively, and each complex is treated as a single kinase that has a gene and mRNA associated with it. The TLR3-dependent transcription factor (TLR3dTF) is a hypothetical transcription factor activated exclusively by the TLR3-TRIF-TRAF6 pathway. To determine possible candidate factors for the TLR3dTF, we focused on transcription factor AP-1 and SP1, which have binding sites in genes being targets of the putative TLR3dTF. To determine specificity of these TFBS, we have performed an additional TFBS search using a dataset of 100 randomly generated 1 kb long DNA sequences. We compared the results to those from our primary dataset, consisting of genes coding for transcription factors of our interest and genes expressed at higher levels in NF-κB and IRF pathways. Results show that AP-1 seems to be non-specific for our primary dataset. The average number of TFBS/per promoter sequence for AP-1 in our dataset is equal to 8.2 (CI = [7.27; 9.13]), which is not significantly lower than average in randomly generated sequences (9.29 TFBS/sequence, CI = [8.74; 9.84]). On the other hand the count of SP1 binding sites significantly differs between primary data set and randomly generated data. In our primary dataset the average count of TFBS/per promoter sequence is equal to 7.4 (CI = [6.42; 8.38]), whereas average for random sequence is equal to 2.4 (CI = [2.13; 2.67]). The highest number of TFBS corresponding to SP1 was found in REL and MAVS genes in human, and MAVS, IKBKB and DDX58 in mouse. The only promoter variant in our dataset, where SP1 TFBS are absent, is one variant of human IRF3 gene promoter. In case of AP-1 there are no genes in our data without binding sites for this transcription factor, with only two promoters containing less than 4TFBS: human NFKBIA and cattle IRF1. These findings are consistent with results from ChIP-Seq data presented in Yang et al. [54], where SP1 enriched chromatin was contained in more rapidly induced genes. Based on the above, our principal TLR3dTF candidate is SP1.

Based on our experimental observations and evolutionary footprint analysis, we include in our model IRF7 as being activated by RelA in presence of dsRNA, is the primary IRF family member inducing transcription of RIG-I in hAECs. For simplicity, we consider IRF7 to be expressed in its activated form. In this situation where dsRNA has strongly activated IKKγ/TBK1, this assumption is defensible.

The model is composed of 83 chemical species and 150 reactions. We present in (Fig. 10) a simplified schematic where mRNAs and inactive forms of the proteins are omitted for clarity. Table 2 includes a summary of principal numerical constants. Pseudocode including all coupled chemical reactions and all constants, the equivalent system of differential equation and a detailed wiring diagram are found in Table ST1 and Figure SF1 in File S1 accordingly.

**Table 2:** Base constant rates used (BCR) to fit the model to experiments.

Description	Parameter	Value
BCR of protein translation	prot_transl_r	5×10^−2^ 1/s
BCR of unregulated protein translation	prot_unreg_transl_r	5×10^−1^ 1/s
BCR of protein degradation	prot_degr_r	10^−5^ 1/s
BCR of unregulated protein degradation	prot_unreg_degr_r	10^−4^ 1/s
BCR of phosphorylated (or active) protein degradation	prot_phosph_degr_r	3×10^−4^ 1/s
BCR of protein binding	prot_bind_r	10^−7^ 1/(#mol s)
BCR of phosphorylation (or activation) of protein	prot_activ_r	10^−8^1/(#mol s)
BCR of protein transition	prot_transition_r	10^−4^ 1/s
BCR of protein dissociation	prot_diss_r	10^−6^ 1/s
BCR of protein nuclear import	prot_import_r	10^−3^ 1/s
BCR of protein nuclear export	prot_export_r	10^−3^ 1/s
BCR of binding to dsRNA	dsRNA_recogn_r	10^−9^ 1/(#mol s)
BCR of dsRNA induced degradation	dsRNA_degr_r	10^−9^ 1/(#mol s)
BCR of gene activation	gene_act_r	10^−7^ 1/(#mol s)
BCR of gene inactivation	gene_inact_r	10^−2^ 1/s
BCR of mRNA transcription (activated gene)	mRNA_transc_r	5×10^−2^ 1/s
BCR of basal mRNA transcription	mRNA_basal_transc_r	10^−3^ 1/s
BCR of mRNA degradation	mRNA_degr_r	10^−4^ 1/s
BCR increment factor of mRNA degradation due to siRNA	siRNA_incr_degr_r	10
Average number of proteins	prot_avg	10^5^ #mol
Cytoplasmic to nucleus ratio	cyto_to_nuc_ratio	5

**Phase 2: Parameter estimation based on time series following dsRNA stimulation.** ***Estimating the number of dsRNA molecules per cell:*** Concentration of dsRNA molecules per cell equals 4 µg/ml. The diameter of hAECs cells was estimated to be 20 µm based on volumetric measurements in Kalita et al. 2010 [11], which resulted in median volume of about 4200 µm^3^ = 4.2×10^−9^ ml. Therefore, if electroporation opened sufficiently many pores in the cell and as a result the concentration of dsRNA in the cell is equal to that in the surrounding medium, this results in 5×10^−7^ µg per cell. Further, the molecular weight of a base pair (bp) is equal to about 650 Daltons (Da), and assuming average length of 300 bp per dsRNA molecule, we obtain circa 200 kDa = 3.24×10^−13^ µg per molecule. This results in approximately 0.5×10^5^ dsRNA molecules per cell. This has to be treated as an upper bound. A more likely figure equals perhaps 1–2×10^4^ dsRNA molecules per cell. As already mentioned, we assumed 2×10^4^ dsRNA molecules per cell.

***Simulations:*** The model was simulated using the bioPN software package [28]. Initial conditions are set to zero number of molecules for all species, with the exception of the number of free (not bound to transcription factor) genes, which is set to 2 copies. The simulation is started at time *t* = −500 hr to assure the system reaches steady state values. At time *t* = 0 hr, electroporation is simulated by inserting, on the average, 2×10^4^ molecules of dsRNA per cell (see the estimation in the next paragraph), which corresponds to the experimental values of 4 µg/ml. To reproduce variability of electroporation, number of molecules, for individual cells, is sampled from a lognormal distribution with coefficient of variation equal to 3. The system is allowed to evolve for 6 hr.

***Fitting model parameters to data:*** Base constant rates (BCR) used to fit the model to experimental results are found in Table 2. The values are selected to obtain physiologically reasonable numbers of mRNAs and proteins. All the chemical reactions of the model are of first or second order. For the second order reactions each BCR needs to be divided by the average number of proteins. As the parameter space is high-dimensional (150 reaction constants parameters), we decided to avoid performing a global optimization procedure as potentially several completely different combinations of parameter values would fit the experimental results. Instead, we started by assigning the corresponding BCR to all the reactions, and applied coefficients to individual reactions in stages in order to achieve an acceptable fit (see further on). The complete set of equations and specific constant rates used are found in the Appendix. In addition, it proved difficult to accurately estimate the absolute counts of molecules in the experimental data. However, we have confidence in the relative molecule counts within time series of each molecular species. Therefore, we fit absolute values simulated by the model to the empirical data scaled in such way that the weighted 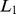 score (average relative absolute difference between simulated and empirical data over all time series) be minimized. The score is computed from the following expression
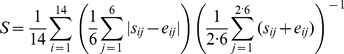
where index 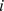 runs over all 14 time series (see Fig. 1), index 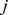 runs over all 6 measurement times (0, 0.5, 1, 2, 4 and 6 hr), and 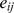 and 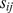 are the empirical and simulated mRNA and protein expressions, respectively. The value of this index for the fits we obtained is equal to 0.171, or average deviation of 17%, which seems to be satisfactory.

***Comparison of observations with model simulations:*** Figure 1 shows both simulated and experimental results for mRNAs of IFNβ, TNFAIP3, RIG-I, and IκBα. Overall, the fit to scaled empirical data is good. Figure 2 shows both simulated and experimental results for the following phosphorylated proteins: cytoplasmic MAVS, IKK2, IKK1, and RIG-I, total (cytoplasmic and nuclear) NF-κB, IκBα, and IRF3; nuclear NF-κB, and total IκBα and IRF3. In all cases the fits seem satisfactory.

**Phase 3: Validation of the model.** The model with parameters estimated (from Phase 2) was programmed to reproduce the results of knockdown experiments.

***Gene knockdown results:*** The results are shown in Fig. 3 using a semi-logarithmic scale to reflect the expected high variability of measurements. Table ST5 in File S1, details the results of significance testing (Welch test) of the effects of knockdowns. As explained in the legend, the minus sign at the p-value denotes reduced expression. Knockdown simulations have been performed by increasing the corresponding mRNA degradation rate by a factor that achieves the same level of down-regulation as shown in the experimental knockdown results for the dsRNA stimulated case (corresponding light-gray bar in Fig. 3). As an example, the top-left panel of Fig. 3 represents the effect of the different knock-downs on RelA. In all cases, dark-gray bars correspond to time 0, while light-gray bars correspond to time 6 hr after stimulation by dsRNA. The first dark-gray bar (Control), has height equal to 1, and heights of the remaining bars are relative to it. Control is a scrambled siRNA which should have no specific effect on RelA expression. The remaining pairs of bars represent the experimental effects, on RelA, of knocking-down RelA, IRF3, RIG-I, and IKKγ. The red horizontal lines depict the simulation prediction of the model. RelA is one of the four genes whose mRNA was knocked-down; therefore, in the second pair of bars, we observe the effect that knocking-down RelA has on itself.

Effect of siRNA on the mRNA expression of RelA (*p*<0.001) and IRF3 (*p* = 0.001) closely agree with the cross inhibition mathematical model.

Effect of RelA siRNA knockdowns in NF-κB dependent genes TNFAIP3/a20 (*p* = 0.002) and NFKBIA/IκBα (*p* = 0.001): all cases are qualitatively matched under the RelA siRNA knockdown. However, the effect shown by experiments by IRF3 siRNA (*p*<0.001 and *p* = 0.003) is not achieved.

Effect of knock-downs in IRF3 dependent genes: only ISG56 has been modeled, and the result shows a qualitative match. Effect on RIG-I expression in murine MEF cells with both IRF3 and IRF7 genes knocked down is depicted in Fig. 3D. RIG-I is down-regulated, which suggests IRF7 may play a key role in RIG-I up-regulation. Compare with RIG-I results in Fig. 3A where it is shown that IRF3 knock-down does not play a role in down-regulating RIG-I (*p* = 0.390).

Effect of knock-downs on IFNβ produces a satisfactory qualitative match. The experimental result shows that RIG-I has a higher impact than IRF3. This may be related to lower specificity of IRF3 siRNA, which cannot completely knock-down its corresponding gene.

**Phase 4: Stochasticity and dose-dependence.** ***Dose-dependence and extrinsic and intrinsic stochasticity:*** The deterministic simulations we carry out, in principle model the behavior of a single cell that absorbs a given number of dsRNA molecules. Moreover the states of all the genes modeled are continuous time functions assuming values between 0 and 2. There are two types of stochasticity which modify this simplistic model. The first of these is the *intrinsic stochasticity*, which arises from the fact that the state of a gene understood as the number of active gene copies is a random variable switching among three values, 0, 1 and 2. In contrast, *extrinsic stochasticity*, identifiable to a large extent with the stochastic distribution of the dsRNA molecules per cell, may play a major role. Therefore, we model the cell population response as an average of 100 trajectories, representing cells with dsRNA molecule numbers distributed lognormally around the mean value of 2×10^4^ molecules of dsRNA with coefficient of variation equal to 3. In Figs. 1 and 2, we depict 30 simulated trajectories as gray lines, their mean as a blue line, and the mean ±1.96 standard errors (95% CI) as a pair of red lines, compared to measurements denoted by solid black circles. Standard errors were computed based on 100 simulations.

***Single-cell trajectories:*** Fig. 6 presents the time series of EGFP-RelA nuclear/cytoplasmic (N/C) ratio in response to 5 µg dsRNA. In some cells, periodic translocations are apparent. A periodogram analysis was performed to decompose the spectral profiles. This analysis mainly indicates noise-distorted periods of approximately 1 or 3 hr. This is in agreement with periodicities exhibited by time series modeling (column 5 in Fig. 6). Fig. 7 presents the time series of N/C ratio at 50 µg of dsRNA. The usual pattern in this case does not involve visually apparent oscillations, but a single translocation, followed by either saturation or partial reversal. However, unexpectedly, the periodogram analysis indicates a superimposed 1–3 hr faint periodicity signal also in this case. Figures 8 and 9 present analogous results for hAEC Strawberry -IRF3 stable cells. Interpretation is also analogous.

In addition, in the Figure SF2 in File S1, we present results of single-cell imaging of non dsRNA-induced cells. No translocation has been observed in these or other replicate experiments.

## Discussion

In this study, we have developed a data-driven model for the integrated epithelial IIR. The airway epithelium includes the first sentinel cells that respond to respiratory viral infection. Our study of the system has employed theoretical development, validated by experimental evidence, to indicate the following features: 1. That NF-κB/RelA and IRF3 pathways cross-inhibit each other; 2. That RIG-I is preferentially coupling to IRF3 signaling; 3. That RIG-I expression is primarily driven by IRF7, in an NF-κB-dependent/IRF3-independent mechanism; and finally 4. Single cell responses are subject to noisy oscillatory behavior. Each of these findings is discussed below.

### Probing the integrated IIR pathway

The integrated epithelial IIR system considered, both experimental and modeled, can be probed in three ways: The first one is by observation of the dynamics of the response of key mRNAs and proteins to a stepwise impulse (adding dsRNA at time t = 0), second by observation of the effects of knockdowns of transcription of key genes on the levels of transcription of other genes, and the third one by exploring the dsRNA dose-dependence of system response. As detailed in the Results, the scaled time series observations were used to estimate parameters of the model. Concerning the knockdown experiments, modeling displays agreement with experiment, validating the model and reinforcing its internal consistency. The exact magnitude of departures depends among other on efficiencies of the knockdowns and unknown interactions which were not taken into account in the model. We consider the agreement achieved very good. Finally, the dose dependence simulations (Fig. 5) result in a dependence interpolating the experimental data.

### Cross talk of RIG-I and TLR3 pathways

The IIR represents a coordinated intracellular response to the presence of invading molecular pathogens. In response to pathogen-encoded molecular patterns, the cell must mount a rapid innate response through a temporally coordinated activation of its major effector arms. How the integrated IIR responds has not been systematically determined. In this paper, we develop and validate a comprehensive model of crosstalk between the RIG-I and TLR3 pathways, both PRRs that play a major role in mediating the early innate immune response to invading viruses. The TLR3 pathway, a pathway that primarily activates the NF-κB transcription factor, has been previously modeled, whereas the RIG-I pathway, a pathway that primarily activates the IRF3 transcription factor, has not yet been modeled. To our knowledge, the crosstalk between these two pathways has not been a subject of systematic analysis.

One of the difficulties in setting up an experimental model of viral infection is that reproducibility may be problematic. For example, active virions do not infect cells synchronously, so that the time of infection is not strictly determined, and also they encode nonstructural proteins that actively disrupt the IIR. Therefore, although modeling the active-virion infections seems more realistic, for practical purposes of understanding pathway kinetics and regulation, we contend that it should be replaced by a more reproducible process. One approach, which we use in this work, is to introduce the pathogen-encoded molecular pattern (in this case, dsRNA) through artificial openings in cell wall made by electric current (e.g., electroporation). Use of this surrogate for modeling a viral infection leads to a reproducible and uniform response, enabling us to understand the kinetics of IIR response in a population that has been stimulated in a synchronous manner. We recognized that our studies are limited because the cellular response is most likely higher than that evoked by active virions. At the dose that is used (4 µg) the response of some molecular pathways may be different than in “natural” conditions. These circumstances have to be considered when analyzing the results of our experiments.

The topology of the IIR has been extensively investigated using focused biochemical experimentation (reviewed in [1]). These studies have shown that the two major signaling effectors, NF-κB and IRF3, are coupled to upstream PRRs through shared intracellular adapters. For example, the activated mitochondrial RIG-I-MAVS PRR signals downstream to NF-κB and IRF3 through both TRAF3 and IKKγ [10]. Both these adapters are necessary for activating IKKα/IKKβ (IKK2 in our model), the rate- limiting kinase controlling NF-κB release and IKKι/TBK1 (IKK1 in our model) the rate-limiting kinase controlling IRF3 translocation. Surprisingly, we observe that the translocation of these two molecules is not synchronous, with IRF3 being translocated more rapidly and transiently than NF-κB (Fig. 2). These findings suggest that additional modulators, perhaps via yet uncharacterized negative feedback loops controlling IRF3 translocation are operative.

Previous work using high density microarrays has shown that the genomic response to the NF-κB signaling arm is mediated by temporally controlled waves of target gene expression [55]. Although the time periods of gene expression observed in this study do not permit us to completely examine the temporal patterns of gene expression induced by the IIR, our data does indicate the existence of delayed response genes. For example, in contrast to the rapid induction of IκBα and TNFAIP3, the induction of IFNβ and RIG-I mRNA are clearly delayed (Fig. 1A). Previous work in the biochemistry of the rapidly responsive IκBα and TNFAIP3 genes has shown that these genes are maintained in an open chromatin configuration and are regulated by a process involving transcriptional elongation [15], [51]. In this mechanism RNA Pol II is bound to the unstimulated promoter. In response to RelA-induced CDK9 recruitment, Pol II is phosphorylated whereupon it acquires processive activity resulting in fully spliced mRNA expression. Consequently these genes are rapidly induced in response to the nuclear presence of activated NF-κB.

By contrast, IFNβ expression is delayed relative to that of the NF-κB-dependent genes. IFNβ encodes a cytokine that functions in a paracrine manner to induce an anti-viral state in adjacent cells [56]. One mechanism for delayed expression may be the requirement for the assembly of a transcription factor complex (an “enhanceosome”) on its promoter prior to its expression. In the resting state, the IFNβ promoter is repressed by the presence of inactive nucleosomes. In response to binding of inducible AP-1 (c-Jun/ATF-2), NF-κB, IRFs, and chromatin remodeling factors, repressive nucleosomes are re-positioned, an event that allows de-repression of the gene [57]. The time required for additional chromatin remodeling may be an explanation for the delayed IFNβ response.

Another delayed response gene is RIG-I, a cytoplasmic protein whose only known function is as a dsRNA PRR. The application of quantitative SRM assays allows us to make interesting observations on the time dependent changes RIG-I protein abundance. Early in the course of response to dsRNA, RIG-I proteins decrease, suggesting that the protein is being actively consumed. This finding is consistent with other biochemical observations that RIG-I undergoes inducible K63-mediated ubiquitylation in response to dsRNA binding [42], a modification that enables its initial association with MAVS; later, K48-linked ubiquitylations mediated by the RNF125 family are responsible to coupling RIG-I and MAVS to the ubiquitin-proteasome degradation pathway [31]. Although more work is required to clarify the relative contributions of stimulus-inducible subcellular compartmentalization, translational regulation and proteasomal degradation, we speculate that RNF125 may mediate the dramatic, parallel reduction in RIG-I and MAVS abundance. Later in the evolution of the dsRNA response, the RIG-I gene is strongly induced, at which point the cytoplasmic levels of RIG-I are replenished. The mechanisms for inducible RIG-I gene expression are not well understood. Previous work has suggested that RIG-I is downstream of the IRF-IFN signaling pathway [58]. However, two of our findings are not consistent with this mechanism. First, RIG-I is induced simultaneously with IFNβ (Fig. 1), a finding that suggests the paracrine effect of IFNβ would be instantaneous and not biologically plausible. Second, siRNA knockdown experiments show that RIG-I is largely independent of IRF3 (Fig. 3). Our predictions therefore are that RIG-I is controlled by a TLR3-dependent transcription factor that is IRF3-independent. To this end, we have discovered that RIG-I induction in IRF7^−/−^ cells is almost completely blocked (Figure 4B). IRF7 expression is IFNβ independent and NF-κB dependent [36], [59]. More work will be required to understand how this NF-κB-IRF7-RIG-I pathway is controlled by dsRNA stimulation.

It is also possible that the observed early depletion of RIG-I, MAVS, IKK1 and IKK2 is caused by the inhibitory effect of dsRNA-dependent protein kinase PKR on mRNA translation. Activated PKR catalyzes the phosphorylation of the subunit of eukaryotic initiation factor 2 (eIF-2α) leading to inhibition of protein synthesis [60]–[62]. However, because these proteins have relatively long half-lives, inhibition of protein synthesis alone is unlikely to be an explanation for our observed findings.

An unanticipated finding from our expression analysis is that NF-κB/RelA and IRF3 are both inducible by dsRNA transfection. These proteins are typically considered to be inert and regulated by post-translational modifications that affect their nuclear and cytoplasmic partitioning. Moreover, our siRNA studies show that NF-κB/RelA and IRF3 gene expression are negatively cross-regulated. The NF-κB and IRF3 signaling pathways are known to be cross-coupled through multiple positive and negative interactions, whose precise temporal interaction is critical for determining the cellular outcome of viral infection. For example, studies in NF-κB -deficient cells have shown that the initial kinetics of the IFNβ response depends on concurrent NF-κB activation [14]. This work has shown that in the absence of NF-κB, the rapid response of IFNβ expression is blunted, reducing the propagation of anti-viral signals in the mucosal surface. This previous result is consistent with the reduced IFNβ expression we observed here in response to RelA siRNA (Fig. 3). Moreover, NF-κB controls expression of STAT1, IRF-1, -5 and -7, transcription factors mediating the downstream IFN auto-amplification loop. Our experiments extend this finding to suggest a weak mutual negative feedback between the two arms of the system, where IRF3 knockdown increases RelA expression and *vice-versa*. This cross-regulatory effect extends to IRF3 and NF-κB -dependent genes as well. Our initial promoter analysis suggests that IRF binding site in present on the RelA promoter, and NF-κB binding site is present on the IRF promoter. Whether these binding sites are functionally important will require further study.

### Approaches to structure and parameter estimation

As mentioned in the Results section above, it is the subject of a debate whether attempts should be made to build a model based on a “rigorous” method such as Maximum Likelihood Estimation or minimization of a performance index, or using an iterative interactive method illustrated here. Girolami [63] argues that it is very difficult to obtain values of the chemical reaction constants which are the parameters of the mathematical model. This uncertainty should be taken into account when the model is used to prevent unproven conclusions about the biological system, or in making overly “optimistic” predictions without understanding the uncertainty of the model. As argued by the same author [63] “Bayesian inferential methodology provides a coherent framework with which to characterize and propagate uncertainty in such mechanistic models”. In the paper involving Girolami's group [64] a similar methodology is applied to ranking of alternative hypothetical topologies of a cell signaling pathway. Similarly as in our experiments, their approach uses measurements of a limited number of biochemical species combined with multiple perturbations. In a more recent work by Chkrebtii et al. [65], a fully Bayesian inferential framework was developed to quantify uncertainty in models defined by general systems of analytically intractable differential equations. The approach was successfully applied to various ordinary and partial differential equation models and to an example characterizing parameter and state uncertainty in a biochemical signaling pathway which incorporates a nonlinear delay-feedback mechanism. These examples are however several times smaller than the system we are considering.

A different philosophy is advocated by Gutenkunst et al. [66], who demonstrated that collective fitting could yield well-constrained predictions, even when it left individual parameters poorly constrained (“sloppy”), with spectrum of sensitivities having eigenvalues distributed over many decades. Mathematical analysis of sloppiness using Vandermonde matrices as in Waterfall et al. [67] and the comparisons in Gutenkunst [66] indicate that models with “sloppy” parameters constitute the rule, not the exception. As they state “prevalence of sloppiness … suggests that modelers should focus on predictions rather than on parameters”. Indirectly, this supports inevitability of iterative interactive approach to identification and estimation in large systems (so-called “tweaking”), which leads to models that may be falsified in the future but provides a welcome reference. In our case, we also took into account modularity of the process, with the dynamics of the NF- κB module being much better known than that of the IRF3 module. We also used evolutionary footprint analysis to evaluate candidate crosstalk agents such as SP1 and AP-1.

### Parameter estimates and the effect of TNFAIP3 knockdown

As it can be noted by inspection of Table ST2 in File S1, the reaction constants in the NF-κB module that fit the time-series data seem quite different from those characterizing dynamics of this module based on its reaction to TNFα stimulation [68]. Without getting into detail, as a rule, the estimates of reaction constants in the present work are lower than those in the in the work cited above. One of the reasons for this is likely that the transients observed under dsRNA stimulation are slower than their counterparts under TNFα stimulation. For example, under standard 10 µg/ml TNFα dose, translocation of RelA into the nucleus occurs within 30 min [68] whereas in our system nuclear RelA reaches peak value after around 1 hour.

In this context it seems interesting to use the model to provide predictions of the effects of knocking down TNFAIP3, which should be propagated down both arms of the system. TNFAIP3 is a negative regulator, so knocking it down is equivalent to not inactivating IKK1 and IKK2. Indeed, as listed in Table 3, a simulated knockdown which reduces expression of the TNFAIP3 gene to about 5% of its normal value at 6 hr following dsRNA stimulation, results in increased expression of IKK1a and IKK2a proteins at 6 hr. However, the results further downstream are different for each arm; while nuclear RelA concentration is increased, there is no significant change in IRF3.

**Table 3:** Modeled effects of theTNFAIP3 knockdown at 6

	Normal	*TNFAIP3* KD
*TNFAIP3* mRNA	700	15
IKK1a	20000	70000
IKK2a	20000	50000
RelAn	60000	80000
IRF3an	10000	10000

This latter finding is related to the fact that to reconcile the model with time series data, we postulate IRF3ii (ii, inert inactive) as a buffer that very slowly transforms into IRF3i (i; inactive). In other words, we are assuming that the recently produced IRF3 (IRF3ii form) needs an unknown number of steps before becoming the mature form (IRF3i form) susceptible to activation. There might exist an analogy between IRF3ii and the pool- NF-κB discovered by Brasier and co-workers [11], [19]. Data also shows IRF3 present in the nucleus at time of stimulation. As it cannot be the active form of IRF3 (otherwise it would activate targets such as IFN and ISG56), we hypothesize that IRF3i can translocate in and out of the nucleus (this allows to fit the time series data). Finally, as IRF3ii to IRF3i transformation is a very slow process, once IRF3i is activated, the amount of IRF3i is depleted very rapidly. TNFAIP3 has a late effect on IKK1a and IKK2a, so even if TNFAIP3 is knocked-down and as a consequence IKK2a stays active, there remains almost no IRF3i to be activated. As a consequence, no effect is noted in the IRF3 arm. The effects described may be specific to the dsRNA stimulation of the system.

Also, in our system, the influence of the TNFAIP3 knockdown does not extend to the expression of RelA or IRF3 target genes. Because of massive stimulation by dsRNA, these genes (such as IκBα and other; not shown) operate at saturation levels. Another reason may be that the IFNbg_NF-κBn_IRF3 an enhanceosome (see the pseudocode in File S1) has half-life of about 10 days, i.e. it very rarely breaks down over the simulation experiment period.

Experimental evidence supporting the influence of TNFAIP3 knockdown on IRF3 activity has been sought. One of the lead studies that shows TNFAIP3 interferes with IRF3 by binding/inhibiting TBK1/IKKε (our IKK2) is Saitoh et al. 2005 [69]. The effect seems to be not very strong considering it was necessary to overexpress TNFAIP3 to show it, and the siRNA knockdown only increased IRF3 activity by 1.5–2-fold.

### Single cell modeling and observations: Influence of extrinsic and intrinsic stochasticity

It has been recognized that variability in dynamic responses of individual biological cells to external stimuli can be caused by a variety of mechanisms two of which are the extrinsic and intrinsic noise. In our system, intrinsic stochasticity seems to be mostly caused by the fact that transcription activation and deactivation is a stochastic process. Extrinsic stochasticity is at least in part accounted for by individual variation of dsRNA dose per cells, which in turn depends on random diffusion of these molecules in the intracellular medium and on the variability of electroporation effects. We consider both effects in our model-based simulations. Inspection of the modeled mRNA and protein levels (grey lines) in Figs. 2A and 3A demonstrates that extrinsic stochasticity accounts for wide variability of the individual cell trajectories around the mean, whereas comparison with Figs. 2B and 3B demonstrates that intrinsic noise contributes qualitatively different phenomena such as oscillations.

How do model predictions compare with single-cell data? Let us consider the nucleus to cytoplasm (N/C) ratio of RelA in Figs. 6 and 7. In low concentrations of dsRNA (5 µg), some cells appear to display 2–3 hr periodicities (cf. the periodograms in Figs. 6 and 7), qualitatively similar to corresponding trajectories (grey lines) in the lower part of the RelA total nuclear graph in Fig. 2B. The range of dynamic effects is wide. In high concentration 50 µg of dsRNA, the pattern involves usually a single major translocation from cytoplasm to nucleus followed by eventual saturation (Fig. 7), qualitatively similar to corresponding trajectories (grey lines) in the upper part of the RelA total nuclear graph in Fig. 2B. However, inspection of periodograms in Fig. 7 proves existence of a faint 2–3 hour periodicity.

Together our studies have illustrated sources of cross-talk in the epithelial IIR. In addition to discovering sources of negative cross-regulation, our studies have predicted and experimentally confirmed the existence of and IRF3-independent, IRF7 dependent linkage between NF-κB and RIG-I expression.

## Supporting Information

File S1 File S1 includes 5 Supplemental Tables and 2 Supplemental Figures. Supplemental Tables are: ST1. System of Ordinary Differential Equations defining the model. ST2. Reaction constants. These reaction constants are the same as in Table ST1 (they are numbered identically). Their dimensions are : 1/(# molecules s) for second-order and 1/s for first-order reactions. ST3. Pseudocode. ST4. TFBS analysis. Table presents counts of TFBS corresponding to IRF family and NF-κB family transcription factors found in promoters of presented genes in four species: cattle (bosTau), mouse (mm), chimpanzee (panTro) and human (hg). Yellow rows correspond to promoter sequences with at least one IRF3 binding site, bright orange rows correspond to sequences containing motifs for all 3 members of IRF family, red cells correspond to sequences with more than 2 binding sites for IRF3. For genes: IRF7, MAVS, IKK1, IKBKB and DDX58 only human and murine promoters were analyzed. ST5. Effect of siRNA knockdown on dsRNA-induced NF-κB/IRF3 gene expressions in A549 cells. Statistical significance of the difference recorded in the knockdown experiment, carried out using the 2-sample, 2-sided t-test (Welch test), corresponding to the bar charts in Figure 3. (A) Comparison of the mRNA-specific siRNA knockdown versus control (nonspecific siRNA), in dsRNA-nonstimulated and dsRNA-stimulated experiment (at 6 hr). (B) Comparison of dsRNAinduced versus dsRNA-noniduced under siRNA knockdown (at 6 hr). Rows: Different knockdowns. Columns: Genes expressed. Supplemental Figures are SF1. Wiring diagram corresponding to the pseudocode and differential equation system. SF2. Snapshots of RelA-specific and IRF3-specific labeling in A549 cells at different times in non dsRNA-induced experiment.
(PDF)
