# Correction: Dynamic Cross Talk Model of the Epithelial Innate Immune Response to Double-Stranded RNA Stimulation: Coordinated Dynamics Emerging from Cell-Level Noise

**DOI:** 10.1371/journal.pone.0103019

**Published:** 2014-07-11

**Authors:** The PLOS ONE Staff

There are errors in the Funding section. The correct funding information is as follows: This work was supported by NIH grants GM086885 (RB, TL, AS, MI, ARB, MK) and AI062885 (ARB), NHLBI-HHSN268201000037C (ARB), UL1TR000071 (ARB) and NIEHS P30 ES006676 (ARB), Polish National Center for Science grant 2011/03/B/NZ2/00281 (TL). MK was partly supported by Polish National Center for Science grant DEC-2012/04/A/ST7/00353 and by EPSRC and the Institute for Advanced Study at the Warwick University, UK, grants supporting his stay as a Fellow of the IAS at the Systems Biology Center at WU in the fall of 2011. The funders had no role in study design, data collection and analysis, decision to publish, or preparation of the manuscript.
