# Supplementary material for: Dynamic Cross Talk Model of the Epithelial Innate Immune Response to Double-Stranded RNA Stimulation: Coordinated Dynamics Emerging from Cell-Level Noise
Source: PLoS One. 2014 Apr 7;9(4):e93396. doi: 10.1371/journal.pone.0093396 (PMC3977818; doi:10.1371/journal.pone.0093396)
Supplement: File S1 — includes 5 Supplemental Tables and 2 Supplemental Figures. Supplemental Tables are: ST1. System of Ordinary Differential Equations defining the model. ST2. Reaction constants. These reaction constants are the same as in Table ST1 (they are numbered identically). Their dimensions are : 1/(# molecules s) for second-order and 1/s for first-order reactions. ST3. Pseudocode. ST4. TFBS analysis. Table presents counts of TFBS corresponding to IRF family and NF-κB family transcription factors found in promoters of presented genes in four species: cattle (bosTau), mouse (mm), chimpanzee (panTro) and human (hg). Yellow rows correspond to promoter sequences with at least one IRF3 binding site, bright orange rows correspond to sequences containing motifs for all 3 members of IRF family, red cells correspond to sequences with more than 2 binding sites for IRF3. For genes: IRF7, MAVS, IKK1, IKBKB and DDX58 only human and murine promoters were analyzed. ST5. Effect of siRNA knockdown on dsRNA-induced NF-κB/IRF3 gene expressions in A549 cells. Statistical significance of the difference recorded in the knockdown experiment, carried out using the 2-sample, 2-sided t-test (Welch test), corresponding to the bar charts in Figure 3. (A) Comparison of the mRNA-specific siRNA knockdown versus control (nonspecific siRNA), in dsRNA-nonstimulated and dsRNA-stimulated experiment (at 6 hr). (B) Comparison of dsRNAinduced versus dsRNA-noniduced under siRNA knockdown (at 6 hr). Rows: Different knockdowns. Columns: Genes expressed. Supplemental Figures are SF1. Wiring diagram corresponding to the pseudocode and differential equation system. SF2. Snapshots of RelA-specific and IRF3-specific labeling in A549 cells at different times in non dsRNA-induced experiment. (PDF) [file pone.0093396.s001.pdf]

DYNAMIC CROSS TALK MODEL OF THE EPITHELIAL INNATE IMMUNE RESPONSE TO DOUBLE-STRANDED RNA STIMULATION: COORDINATED DYNAMICS EMERGING FROM CELL-LEVEL NOISE

Roberto Bertolusso, Bing Tian, Yingxin Zhao, Leoncio Vergara, Aqeeb Sabree, Marta Iwanaszko, Tomasz Lipniacki, Allan R Brasier, and Marek Kimmel

SUPPLEMENT FILE

Table of Contents

I. **Supplemental Tables**
  1. ST1: System of Ordinary Differential Equations defining the model
  2. ST2: Reaction constants
  3. ST3: Pseudocode
  4. ST4: TFBS analysis
  5. ST5: Effect of siRNA knockdown on dsRNA-induced NF-kB/IRF3 gene expressions in A549 cells

II. **Supplemental Figures**
  1. SF1: Wiring diagram corresponding to the pseudocode and differential equation system.
  2. SF2: Snapshots of RelA-specific and IRF3-specific labeling in A549 cells at different times in non dsRNA-induced experiment

DYNAMIC CROSS TALK MODEL OF THE EPITHELIAL INNATE IMMUNE RESPONSE TO DOUBLE-STRANDED RNA STIMULATION: COORDINATED DYNAMICS EMERGING FROM CELL-LEVEL NOISE

Roberto Bertolusso, Bing Tian, Yingxin Zhao, Leoncio Vergara, Aqeeb Sabree, Marta Iwanaszko, Tomasz Lipniacki, Allan R Brasier, and Marek Kimmel

ST1: **System of Ordinary Differential Equations** defining the model

$$\dot{\text{dsRNA}} = -h_1 \cdot \text{dsRNA} \cdot \text{RIGI} - h_{16} \cdot \text{dsRNA} \cdot \text{TLR3i} \tag{1}$$

$$\dot{\text{RIGIg}} = -h_{39} \cdot \text{RIGIg} \cdot \text{IRF7an} + h_{40} \cdot \text{RIGIg\_IRF7an} \tag{2}$$

$$\dot{\text{RIGIg\_IRF7an}} = h_{39} \cdot \text{RIGIg} \cdot \text{IRF7an} - h_{40} \cdot \text{RIGIg\_IRF7an} \tag{3}$$

$$\dot{\text{RIGIt}} = h_{41} \cdot \text{RIGIg\_IRF7an} + h_{42} \cdot \text{RIGIg} - h_{43} \cdot \text{RIGIt} \tag{4}$$

$$\dot{\text{RIGI}} = -h_2 \cdot \text{dsRNA} \cdot \text{RIGI} + h_{44} \cdot \text{RIGIt} - h_{45} \cdot \text{RIGI} \tag{5}$$

$$\dot{\text{RIGIub}} = h_2 \cdot \text{dsRNA} \cdot \text{RIGI} - h_3 \cdot \text{RIGIub} - h_4 \cdot \text{RIGIub} \cdot \text{MAVS} \tag{6}$$

$$\dot{\text{MAVSg}} = -h_{46} \cdot \text{MAVSg} \cdot \text{TLR3dTFan} + h_{47} \cdot \text{MAVSg\_TLR3dTFan} \tag{7}$$

$$\dot{\text{MAVSg\_TLR3dTFan}} = h_{46} \cdot \text{MAVSg} \cdot \text{TLR3dTFan} - h_{47} \cdot \text{MAVSg\_TLR3dTFan} \tag{8}$$

$$\dot{\text{MAVSt}} = h_{48} \cdot \text{MAVSg\_TLR3dTFan} + h_{49} \cdot \text{MAVSg} - h_{50} \cdot \text{MAVSt} \tag{9}$$

$$\dot{\text{MAVS}} = -h_4 \cdot \text{RIGIub} \cdot \text{MAVS} + h_{51} \cdot \text{MAVSt} - h_{52} \cdot \text{MAVS} \tag{10}$$

$$\dot{\text{RIGI\_MAVS}} = h_4 \cdot \text{RIGIub} \cdot \text{MAVS} - h_5 \cdot \text{RIGI\_MAVS} - h_6 \cdot \text{RIGI\_MAVS} \cdot \text{TRAF3} \tag{11}$$

$$\dot{\text{TRAF3g}} = 0 \tag{12}$$

$$\dot{\text{TRAF3t}} = h_{135} \cdot \text{TRAF3g} - h_{136} \cdot \text{TRAF3t} \tag{13}$$

$$\dot{\text{TRAF3}} = -h_6 \cdot \text{RIGI\_MAVS} \cdot \text{TRAF3} + h_{137} \cdot \text{TRAF3t} - h_{138} \cdot \text{TRAF3} \tag{14}$$

$$\dot{\text{RIGI\_MAVS\_TRAF3}} = h_6 \cdot \text{RIGI\_MAVS} \cdot \text{TRAF3} - h_7 \cdot \text{RIGI\_MAVS\_TRAF3} \tag{15}$$

$$\dot{\text{IKK1g}} = -h_{53} \cdot \text{IKK1g} \cdot \text{TLR3dTFan} + h_{54} \cdot \text{IKK1g\_TLR3dTFan} \tag{16}$$

$$\dot{\text{IKK1g\_TLR3dTFan}} = h_{53} \cdot \text{IKK1g} \cdot \text{TLR3dTFan} - h_{54} \cdot \text{IKK1g\_TLR3dTFan} \tag{17}$$

$$\dot{\text{IKK1t}} = h_{55} \cdot \text{IKK1g\_TLR3dTFan} + h_{56} \cdot \text{IKK1g} - h_{57} \cdot \text{IKK1t} \tag{18}$$

$$\dot{\text{IKK1i}} = -h_8 \cdot \text{RIGI\_MAVS\_TRAF3} \cdot \text{IKK1i} + h_{58} \cdot \text{IKK1t} - h_{59} \cdot \text{IKK1i} \tag{19}$$

$$\dot{\text{IKK1a}} = h_8 \cdot \text{RIGI\_MAVS\_TRAF3} \cdot \text{IKK1i} - h_9 \cdot \text{IKK1a} - h_{10} \cdot \text{IKK1a} \cdot \text{TNFAIP3} \tag{20}$$

$$\dot{\text{IRF3g}} = -h_{67} \cdot \text{IRF3g} \cdot \text{RelAn} + h_{68} \cdot \text{IRF3g\_RelAn} - h_{69} \cdot \text{IRF3g} \cdot \text{TLR3dTFan} + h_{70} \cdot \text{IRF3g\_TLR3dTFan} \tag{21}$$

$$\dot{\text{IRF3g\_TLR3dTFan}} = h_{69} \cdot \text{IRF3g} \cdot \text{TLR3dTFan} - h_{70} \cdot \text{IRF3g\_TLR3dTFan} \tag{22}$$

$$\dot{\text{IRF3g\_RelAn}} = h_{67} \cdot \text{IRF3g} \cdot \text{RelAn} - h_{68} \cdot \text{IRF3g\_RelAn} \tag{23}$$

$$\dot{\text{IRF3t}} = h_{71} \cdot \text{IRF3g\_TLR3dTFan} + h_{72} \cdot \text{IRF3g} - h_{73} \cdot \text{IRF3t} \tag{24}$$

$$\dot{\text{IRF3ii}} = h_{74} \cdot \text{IRF3t} - h_{75} \cdot \text{IRF3ii} - h_{76} \cdot \text{IRF3ii} \tag{25}$$

$$\dot{\text{IRF3i}} = -h_{11} \cdot \text{IKK1a} \cdot \text{IRF3i} + h_{76} \cdot \text{IRF3ii} - h_{77} \cdot \text{IRF3i} - h_{78} \cdot \text{IRF3i} + h_{79} \cdot \text{IRF3in} \tag{26}$$

$$\dot{\text{IRF3in}} = h_{78} \cdot \text{IRF3i} - h_{79} \cdot \text{IRF3in} - h_{80} \cdot \text{IRF3in} \tag{27}$$

$$\dot{\text{IRF3a}} = h_{11} \cdot \text{IKK1a} \cdot \text{IRF3i} - h_{12} \cdot \text{IRF3a} - h_{13} \cdot \text{IRF3a} + h_{14} \cdot \text{IRF3an} \tag{28}$$

$$\begin{aligned}\dot{\text{IRF3an}} = h_{13} \cdot \text{IRF3a} - h_{14} \cdot \text{IRF3an} - h_{15} \cdot \text{IRF3an} - h_{81} \cdot \text{IRF3an} \cdot \text{RelAg} + \\ h_{82} \cdot \text{RelAg\_IRF3an} - h_{117} \cdot \text{IRF3an} \cdot \text{ISG56g} + \\ h_{118} \cdot \text{ISG56g\_IRF3an} - h_{125} \cdot \text{IRF3an} \cdot \text{IFNbg\_RelAn} + \\ h_{126} \cdot \text{IFNbg\_RelAn\_IRF3an}\end{aligned} \tag{29}$$

$$\dot{\text{IRF7g}} = -h_{110} \cdot \text{I(dsRNA)} \cdot \text{IRF7g} \cdot \text{RelAn} + h_{111} \cdot \text{IRF7g\_RelAn} \tag{30}$$

$$\dot{\text{IRF7g\_RelAn}} = h_{110} \cdot \text{I(dsRNA)} \cdot \text{IRF7g} \cdot \text{RelAn} - h_{111} \cdot \text{IRF7g\_RelAn} \tag{31}$$

$$\dot{\text{IRF7t}} = h_{112} \cdot \text{IRF7g\_RelAn} - h_{113} \cdot \text{IRF7t} \tag{32}$$

$$\dot{\text{IRF7a}} = h_{114} \cdot \text{IRF7t} - h_{115} \cdot \text{IRF7a} \tag{33}$$

$$\dot{\text{IRF7an}} = -h_{39} \cdot \text{RIGIg} \cdot \text{IRF7an} + h_{40} \cdot \text{RIGIg\_IRF7an} + h_{115} \cdot \text{IRF7a} - h_{116} \cdot \text{IRF7an} \tag{34}$$

$$\dot{\text{TLR3g}} = 0 \tag{35}$$

$$\dot{\text{TLR3t}} = h_{131} \cdot \text{TLR3g} - h_{132} \cdot \text{TLR3t} \tag{36}$$

$$\dot{\text{TLR3i}} = -h_{17} \cdot \text{dsRNA} \cdot \text{TLR3i} + h_{133} \cdot \text{TLR3t} - h_{134} \cdot \text{TLR3i} \tag{37}$$

$$\dot{\text{TLR3a}} = h_{17} \cdot \text{dsRNA} \cdot \text{TLR3i} - h_{18} \cdot \text{TLR3a} - h_{19} \cdot \text{TLR3a} \cdot \text{TRIF} \tag{38}$$

$$\dot{\text{TRIFg}} = 0 \tag{39}$$

$$\dot{\text{TRIFt}} = h_{143} \cdot \text{TRIFg} - h_{144} \cdot \text{TRIFt} \tag{40}$$

$$\dot{\text{TRIF}} = -h_{19} \cdot \text{TLR3a} \cdot \text{TRIF} + h_{145} \cdot \text{TRIFt} - h_{146} \cdot \text{TRIF} \tag{41}$$

$$\dot{\text{TLR3\_TRIF}} = h_{19} \cdot \text{TLR3a} \cdot \text{TRIF} - h_{20} \cdot \text{TLR3\_TRIF} - h_{21} \cdot \text{TLR3\_TRIF} \cdot \text{TRAF6} \tag{42}$$

$$\dot{\text{TRAF6g}} = 0 \tag{43}$$

$$\dot{\text{TRAF6t}} = h_{139} \cdot \text{TRAF6g} - h_{140} \cdot \text{TRAF6t} \tag{44}$$

$$\dot{\text{TRAF6}} = -h_{21} \cdot \text{TLR3\_TRIF} \cdot \text{TRAF6} + h_{141} \cdot \text{TRAF6t} - h_{142} \cdot \text{TRAF6} \tag{45}$$

$$\dot{\text{TLR3\_TRIF\_TRAF6}} = h_{21} \cdot \text{TLR3\_TRIF} \cdot \text{TRAF6} - h_{22} \cdot \text{TLR3\_TRIF\_TRAF6} \tag{46}$$

$$\dot{\text{IKK2g}} = -h_{60} \cdot \text{IKK2g} \cdot \text{TLR3dTFan} + h_{61} \cdot \text{IKK2g\_TLR3dTFan} \tag{47}$$

$$\dot{\text{IKK2g\_TLR3dTFan}} = h_{60} \cdot \text{IKK2g} \cdot \text{TLR3dTFan} - h_{61} \cdot \text{IKK2g\_TLR3dTFan} \tag{48}$$

$$\dot{\text{IKK2t}} = h_{62} \cdot \text{IKK2g\_TLR3dTFan} + h_{63} \cdot \text{IKK2g} - h_{64} \cdot \text{IKK2t} \tag{49}$$

$$\dot{\text{IKK2i}} = -h_{26} \cdot \text{TLR3\_TRIF\_TRAF6} \cdot \text{IKK2i} + h_{65} \cdot \text{IKK2t} - h_{66} \cdot \text{IKK2i} \tag{50}$$

$$\dot{\text{IKK2a}} = h_{26} \cdot \text{TLR3\_TRIF\_TRAF6} \cdot \text{IKK2i} - h_{27} \cdot \text{IKK2a} - h_{28} \cdot \text{IKK2a} \cdot \text{TNFAIP3} \tag{51}$$

$$\dot{\text{TLR3dTFg}} = 0 \tag{52}$$

$$\dot{\text{TLR3dTFt}} = h_{147} \cdot \text{TLR3dTFg} - h_{148} \cdot \text{TLR3dTFt} \tag{53}$$

$$\dot{\text{TLR3dTFi}} = -h_{23} \cdot \text{TLR3\_TRIF\_TRAF6} \cdot \text{TLR3dTFi} + h_{149} \cdot \text{TLR3dTFt} - h_{150} \cdot \text{TLR3dTFi} \tag{54}$$

$$\dot{\text{TLR3dTFa}} = h_{23} \cdot \text{TLR3\_TRIF\_TRAF6} \cdot \text{TLR3dTFi} - h_{24} \cdot \text{TLR3dTFa} \tag{55}$$

$$\begin{aligned}\dot{\text{TLR3dTFan}} = h_{24} \cdot \text{TLR3dTFa} - h_{25} \cdot \text{TLR3dTFan} - h_{46} \cdot \text{MAVSg} \cdot \text{TLR3dTFan} + \\ h_{47} \cdot \text{MAVSg\_TLR3dTFan} - h_{53} \cdot \text{IKK1g} \cdot \text{TLR3dTFan} + \\ h_{54} \cdot \text{IKK1g\_TLR3dTFan} - h_{60} \cdot \text{IKK2g} \cdot \text{TLR3dTFan} + \\ h_{61} \cdot \text{IKK2g\_TLR3dTFan} - h_{69} \cdot \text{IRF3g} \cdot \text{TLR3dTFan} + \\ h_{70} \cdot \text{IRF3g\_TLR3dTFan} - h_{83} \cdot \text{TLR3dTFan} \cdot \text{RelAg} + \\ h_{84} \cdot \text{RelAg\_TLR3dTFan}\end{aligned} \tag{56}$$

$$\dot{\text{RelAg}} = -h_{81} \cdot \text{IRF3an} \cdot \text{RelAg} + h_{82} \cdot \text{RelAg\_IRF3an} - h_{83} \cdot \text{TLR3dTFan} \cdot \text{RelAg} + h_{84} \cdot \text{RelAg\_TLR3dTFan} \quad (57)$$

$$\dot{\text{RelAg\_TLR3dTFan}} = h_{83} \cdot \text{TLR3dTFan} \cdot \text{RelAg} - h_{84} \cdot \text{RelAg\_TLR3dTFan} \quad (58)$$

$$\dot{\text{RelAg\_IRF3an}} = h_{81} \cdot \text{IRF3an} \cdot \text{RelAg} - h_{82} \cdot \text{RelAg\_IRF3an} \quad (59)$$

$$\dot{\text{RelAt}} = h_{85} \cdot \text{RelAg\_TLR3dTFan} + h_{86} \cdot \text{RelAg} - h_{87} \cdot \text{RelAt} \quad (60)$$

$$\dot{\text{RelAi}} = h_{88} \cdot \text{RelAt} - h_{89} \cdot \text{RelAi} - h_{90} \cdot \text{RelAi} + h_{91} \cdot \text{RelA} - h_{92} \cdot \text{IKK2a} \cdot \text{RelAi} \quad (61)$$

$$\dot{\text{RelA}} = h_{31} \cdot \text{IKK2a} \cdot \text{RelA\_IkBa} - h_{32} \cdot \text{RelA} \cdot \text{IkBa} + h_{33} \cdot \text{RelA\_IkBa} - h_{34} \cdot \text{RelA} + h_{90} \cdot \text{RelAi} - h_{91} \cdot \text{RelA} + h_{92} \cdot \text{IKK2a} \cdot \text{RelAi} - h_{93} \cdot \text{RelA} \quad (62)$$

$$\dot{\text{RelAn}} = h_{34} \cdot \text{RelA} - h_{35} \cdot \text{RelAn} \cdot \text{IkBan} - h_{67} \cdot \text{IRF3g} \cdot \text{RelAn} + h_{68} \cdot \text{IRF3g\_RelAn} + -h_{94} \cdot \text{RelAn} - h_{95} \cdot \text{RelAn} \cdot \text{TNFAIP3g} - h_{101} \cdot \text{RelAn} \cdot \text{IkBag} + -h_{110} \cdot \text{dsRNA} \cdot \text{IRF7g} \cdot \text{RelAn} + h_{111} \cdot \text{IRF7g\_RelAn} - h_{123} \cdot \text{RelAn} \cdot \text{IFNbg} + h_{126} \cdot \text{IFNbg\_RelAn\_IRF3an} \quad (63)$$

$$\dot{\text{IkBag}} = -h_{101} \cdot \text{RelAn} \cdot \text{IkBag} + h_{102} \cdot \text{IkBag\_RelAn} \cdot \text{IkBan} \quad (64)$$

$$\dot{\text{IkBag\_RelAn}} = h_{101} \cdot \text{RelAn} \cdot \text{IkBag} - h_{102} \cdot \text{IkBag\_RelAn} \cdot \text{IkBan} \quad (65)$$

$$\dot{\text{IkBat}} = h_{103} \cdot \text{IkBag\_RelAn} - h_{104} \cdot \text{IkBat} \quad (66)$$

$$\dot{\text{IkBa}} = -h_{29} \cdot \text{IKK2a} \cdot \text{IkBa} - h_{32} \cdot \text{RelA} \cdot \text{IkBa} + h_{33} \cdot \text{RelA\_IkBa} + h_{105} \cdot \text{IkBat} - h_{106} \cdot \text{IkBa} + -h_{107} \cdot \text{IkBa} + h_{109} \cdot \text{IkBan} \quad (67)$$

$$\dot{\text{IkBan}} = -h_{35} \cdot \text{RelAn} \cdot \text{IkBan} - h_{96} \cdot \text{IkBan} \cdot \text{TNFAIP3g\_RelAn} - h_{102} \cdot \text{IkBag\_RelAn} \cdot \text{IkBan} + h_{107} \cdot \text{IkBa} - h_{108} \cdot \text{IkBan} - h_{109} \cdot \text{IkBan} - h_{124} \cdot \text{IkBan} \cdot \text{IFNbg\_RelAn} \quad (68)$$

$$\dot{\text{RelA\_IkBa}} = -h_{30} \cdot \text{RelA\_IkBa} - h_{31} \cdot \text{IKK2a} \cdot \text{RelA\_IkBa} + h_{32} \cdot \text{RelA} \cdot \text{IkBa} - h_{33} \cdot \text{RelA\_IkBa} + -h_{37} \cdot \text{RelA\_IkBa} + h_{38} \cdot \text{RelAn\_IkBan} \quad (69)$$

$$\dot{\text{RelAn\_IkBan}} = h_{35} \cdot \text{RelAn} \cdot \text{IkBan} - h_{36} \cdot \text{RelAn\_IkBan} + h_{37} \cdot \text{RelA\_IkBa} - h_{38} \cdot \text{RelAn\_IkBan} + h_{96} \cdot \text{IkBan} \cdot \text{TNFAIP3g\_RelAn} + h_{102} \cdot \text{IkBag\_RelAn} \cdot \text{IkBan} + h_{124} \cdot \text{IkBan} \cdot \text{IFNbg\_RelAn} \quad (70)$$

$$\dot{\text{TNFAIP3g}} = -h_{95} \cdot \text{RelAn} \cdot \text{TNFAIP3g} + h_{96} \cdot \text{IkBan} \cdot \text{TNFAIP3g\_RelAn} \quad (71)$$

$$\dot{\text{TNFAIP3g\_RelAn}} = h_{95} \cdot \text{RelAn} \cdot \text{TNFAIP3g} - h_{96} \cdot \text{IkBan} \cdot \text{TNFAIP3g\_RelAn} \quad (72)$$

$$\dot{\text{TNFAIP3t}} = h_{97} \cdot \text{TNFAIP3g\_RelAn} - h_{98} \cdot \text{TNFAIP3t} \quad (73)$$

$$\dot{\text{TNFAIP3}} = h_{99} \cdot \text{TNFAIP3t} - h_{100} \cdot \text{TNFAIP3} \quad (74)$$

$$\dot{\text{IFNbg}} = -h_{123} \cdot \text{RelAn} \cdot \text{IFNbg} + h_{124} \cdot \text{IkBan} \cdot \text{IFNbg\_RelAn} + h_{126} \cdot \text{IFNbg\_RelAn\_IRF3an} \quad (75)$$

$$\dot{\text{IFNbg\_RelAn}} = h_{123} \cdot \text{RelAn} \cdot \text{IFNbg} - h_{124} \cdot \text{IkBan} \cdot \text{IFNbg\_RelAn} - h_{125} \cdot \text{IRF3an} \cdot \text{IFNbg\_RelAn} \quad (76)$$

$$\dot{\text{IFNbg\_RelAn\_IRF3an}} = h_{125} \cdot \text{IRF3an} \cdot \text{IFNbg\_RelAn} - h_{126} \cdot \text{IFNbg\_RelAn\_IRF3an} \quad (77)$$

$$\dot{\text{IFNbt}} = h_{127} \cdot \text{IFNbg\_RelAn\_IRF3an} - h_{128} \cdot \text{IFNbt} \quad (78)$$

$$\dot{\text{IFNb}} = h_{129} \cdot \text{IFNbt} - h_{130} \cdot \text{IFNb} \quad (79)$$

$$\dot{\text{ISG56g}} = -h_{117} \cdot \text{IRF3an} \cdot \text{ISG56g} + h_{118} \cdot \text{ISG56g\_IRF3an} \quad (80)$$

$$\dot{\text{ISG56g\_IRF3an}} = h_{117} \cdot \text{IRF3an} \cdot \text{ISG56g} - h_{118} \cdot \text{ISG56g\_IRF3an} \quad (81)$$

$$\dot{\text{ISG56t}} = h_{119} \cdot \text{ISG56g\_IRF3an} - h_{120} \cdot \text{ISG56t} \quad (82)$$

$$\dot{\text{ISG56}} = h_{121} \cdot \text{ISG56t} - h_{122} \cdot \text{ISG56} \quad (83)$$

Supplemental Table ST2: Reaction constants. These reaction constants are the same as in Table ST1 (they are numbered identically). Their dimensions are : 1 /(# molecules s) for second-order and 1/s for first-order reactions.

"reaction.name"	constant.value
1 "dsRNA_db_RIGI"	1.00E-09
2 "RIGI_ubiq"	1.00E-09
3 "RIGIub_d"	3.00E-04
4 "RIGI_b_MAVS"	1.00E-07
5 "RIGI_MAVS_d"	3.00E-04
6 "RIGI_MAVS_b_TRAF3"	1.00E-07
7 "RIGI_MAVS_TRAF3_d"	3.00E-04
8 "IKK1i_a"	1.00E-08
9 "IKK1a_d"	3.00E-04
10 "IKK1a_db_A20"	3.00E-09
11 "IRF3i_a"	1.00E-08
12 "IRF3a_deg"	3.00E-04
13 "IRF3a_i"	0.001
14 "IRF3an_e"	0.001
15 "IRF3an_d"	3.00E-04
16 "dsRNA_db_TLR3i"	1.00E-09
17 "TLR3i_act"	1.00E-09
18 "TLR3a_d"	3.00E-04
19 "TLR3_b_TRIF"	1.00E-07
20 "TLR3_TRIF_d"	3.00E-04
21 "TLR3_TRIF_b_TRAF6"	1.00E-07
22 "TLR3_TRIF_TRAF6_d"	3.00E-04
23 "TLR3dTFi_a"	1.00E-08
24 "TLR3dTFa_imp"	0.001
25 "TLR3dTFan_d"	0.00015
26 "IKK2i_a"	1.00E-08
27 "IKK2a_d"	3.00E-04
28 "IKK2a_db_A20"	1.5e-09
29 "IkBa_d"	3.00E-06
30 "RelA_IkBa_d"	1.00E-05
31 "RelA_IkBa_dis"	3.00E-06
32 "RelA_b_IkBa"	1.00E-07
33 "RelA_IkBa_sd"	1.00E-06
34 "RelA_i"	0.001
35 "RelAn_b_IkBan"	3.42e-07
36 "RelAn_IkBan_d"	1.00E-05
37 "RelA_IkBa_i"	1.00E-04
38 "RelAn_IkBan_e"	0.001
39 "RIGIg_a"	2.00E-09
40 "RIGIg_IRF7an_i"	2.00E-05
41 "RIGIt_t"	0.1
42 "RIGIt_bt"	0.001
43 "RIGIt_d"	1.00E-04
76 "IRF3ii_to_IRF3i"	1.00E-04
77 "IRF3i_d"	1.00E-05
78 "IRF3i_imp"	1.00E-04
79 "IRF3i_exp"	0.001
80 "IRF3in_d"	1.00E-05
81 "RelAg_b_IRF3an"	2.00E-07
82 "RelAg_IRF3an_d"	0.001
83 "RelAg_a"	1.00E-07
84 "RelAg_TLR3dTFan_i"	0.01
85 "RelAt_t"	0.05
86 "RelAt_bt"	0.001
87 "RelAt_d"	1.00E-04
88 "RelAi_s"	0.05
89 "RelAi_d"	1.00E-05
90 "RelAi_to_RelA"	1.00E-05
91 "RelA_to_RelAi"	1.00E-05
92 "RelAi_to_RelA_by_IKK2a"	1.00E-09
93 "RelA_d"	1.00E-05
94 "RelAn_d"	1.00E-05
95 "A20g_a"	8.00E-09
96 "A20g_RelAn_i"	2.00E-07
97 "A20t_t"	0.05
98 "A20t_d"	1.00E-04
99 "A20_s"	0.05
100 "A20_d"	1.00E-05
101 "IkBag_a"	2.5e-08
102 "IkBag_RelAn_i"	2.5e-07
103 "IkBat_t"	0.25
104 "IkBat_d"	4.00E-04
105 "IkBa_s"	0.5
106 "IkBa_d"	1.00E-04
107 "IkBa_imp"	0.001
108 "IkBan_d"	1.00E-04
109 "IkBa_exp"	5.00E-04
110 "IRF7g_a"	3.00E-07
111 "IRF7g_RelAn_i"	0.01
112 "IRF7t_t"	0.05
113 "IRF7t_d"	1.00E-04
114 "IRF7a_s"	0.05
115 "IRF7a_imp"	0.001
116 "IRF7an_d"	0.00015
117 "ISG56g_a"	1.00E-06
118 "ISG56g_IRF3an_i"	0.01

44 "RIGI_s"	0.05	119 "ISG56t_t"	0.05
45 "RIGI_d"	1.00E-05	120 "ISG56t_d"	1.00E-04
46 "MAVSg_a"	1.00E-09	121 "ISG56_s"	0.05
47 "MAVSg_TLR3dTFab_i"	1.00E-04	122 "ISG56_d"	1.00E-05
48 "MAVSt_t"	0.075	123 "IFNbg_a_RelAn"	7.5e-08
49 "MAVSt_bt"	0.001	124 "IFNbg_RelAn_i_IkBa"	2.00E-06
50 "MAVSt_d"	1.00E-04	125 "IFNbg_RelAn_a_IRF3an"	2.00E-09
51 "MAVS_s"	0.05	126 "IFNbg_RelAn_IRF3an_i"	1.00E-05
52 "MAVS_d"	1.00E-05	127 "IFNbt_t"	0.25
53 "IKK1g_a"	3.00E-07	128 "IFNbt_d"	1.00E-04
54 "IKK1g_TLR3dTFan_i"	0.01	129 "IFNb_s"	0.05
55 "IKK1t_t"	0.05	130 "IFNb_d"	1.00E-05
56 "IKK1t_bt"	0.001	131 "TLR3t_bt"	0.001
57 "IKK1t_d"	1.00E-04	132 "TLR3t_d"	1.00E-04
58 "IKK1i_s"	0.05	133 "TLR3i_s"	0.5
59 "IKK1i_d"	1.00E-05	134 "TLR3i_d"	1.00E-04
60 "IKK2g_a"	3.00E-07	135 "TRAF3t_bt"	0.001
61 "IKK2g_TLR3dTFan_i" x	0.05	136 "TRAF3t_d"	1.00E-04
62 "IKK2t_t"	0.05	137 "TRAF3_s"	0.5
63 "IKK2t_bt"	0.001	138 "TRAF3_d"	1.00E-04
64 "IKK2t_d"	1.00E-04	139 "TRAF6t_bt"	0.001
65 "IKK2i_s"	0.05	140 "TRAF6t_d"	1.00E-04
66 "IKK2i_d"	1.00E-05	141 "TRAF6_s"	0.5
67 "IRF3g_b_RelAn"	5.00E-07	142 "TRAF6_d"	1.00E-04
68 "IRF3g_RelAn_d"	0.01	143 "TRIFt_bt"	0.001
69 "IRF3g_a"	3.00E-07	144 "TRIFt_d"	1.00E-04
70 "IRF3g_TLR3dTFan_i"	0.01	145 "TRIF_s"	0.5
71 "IRF3t_t"	0.05	146 "TRIF_d"	1.00E-04
72 "IRF3t_bt"	0.001	147 "TLR3dTFt_bt"	0.001
73 "IRF3t_d"	1.00E-04	148 "TLR3dTFt_d"	1.00E-04
74 "IRF3ii_s"	0.05	149 "TLR3dTFi_s"	0.5
75 "IRF3ii_d"	1.00E-05	150 "TLR3dTFi_d"	1.00E-04

Supplemental Table ST3: Pseudocode.

******* General constants and parameters *******

```
prot_avg          = 1e5
cyto_to_nuc_ratio   = 3.42
siRNA_incr_degr_r   = 1e1

prot_transl_r      = 5e-2
prot_unreg_transl_r = 5e-1

prot_degr_r        = 1e-5
prot_unreg_degr_r   = 1e-4
prot_phosph_degr_r  = 3e-4

prot_bind_r        = 1e-2
prot_activ_r       = 1e-3
prot_transition_r   = 1e-4

prot_diss_r        = 1e-6
prot_import_r      = 1e-3
prot_export_r      = 1e-3

dsRNA_recogn_r     = 1e-4 * 5e5 / dsRNA.mean
dsRNA_degr_r       = 1e-4

gene_act_r         = 1e-2
gene_inact_r       = 1e-2

mRNA_transc_r      = 5e-2
mRNA_basal_transc_r = 1e-3
mRNA_degr_r        = 1e-4

RelAn_IRF3an_ratio  = 2
```

******** Start of model definition ********

******* Left arm recognition of dsRNA and RIGI_MAVS_TRAF3 complex formation *******

```
------ dsRNA degradation induced by RIGI:
Ch.E.: RIGI + dsRNA ---> RIGI
Rt.C.: dsRNA_degr_r / prot_avg

------ RIGI ubiquitylation:
Ch.E.: dsRNA + RIGI ---> dsRNA + RIGIub
Rt.C.: dsRNA_recogn_r / prot_avg

------ RIGIub degradation:
Ch.E.: RIGIub ---> 0
```

Rt.C.: prot_phosph_degr_r

------ RIGIub and MAVS binding:
Ch.E.: RIGIub + MAVS ---> RIGI_MAVS
Rt.C.: prot_bind_r / prot_avg

------ RIGI_MAVS degradation:
Ch.E.: RIGI_MAVS ---> 0
Rt.C.: prot_phosph_degr_r

------ RIGI_MAVS and TRAF3 binding:
Ch.E.: RIGI_MAVS + TRAF3 ---> RIGI_MAVS_TRAF3
Rt.C.: prot_bind_r / prot_avg

------ RIGI_MAVS_TRAF3 degradation:
Ch.E.: RIGI_MAVS_TRAF3 ---> 0
Rt.C.: prot_phosph_degr_r

******* IKK1 (TBK1/IKKe) activation by RIGI_MAVS_TRAF3 and regulation by A20 *******

------ IKK1i activation:
Ch.E.: RIGI_MAVS_TRAF3 + IKK1i ---> RIGI_MAVS_TRAF3 + IKK1a
Rt.C.: prot_activ_r / prot_avg

------ IKK1a degradation:
Ch.E.: IKK1a ---> 0
Rt.C.: prot_phosph_degr_r

------ IKK1a degradation due to A20:
Ch.E.: A20 + IKK1a ---> A20
Rt.C.: prot_phosph_degr_r / prot_avg

******* IRF3 activation by IKK1 and nuclear translocation *******

------ IRF3i activation:
Ch.E.: IKK1a + IRF3i ---> IKK1a + IRF3a
Rt.C.: prot_activ_r / prot_avg

------ IRF3a degradation:
Ch.E.: IRF3a ---> 0
Rt.C.: prot_phosph_degr_r

------ IRF3a import:
Ch.E.: IRF3a ---> IRF3an
Rt.C.: prot_import_r

------ IRF3a export:

Ch.E.: IRF3an ---> IRF3a
Rt.C.: prot_export_r

------ IRF3an degradation:
Ch.E.: IRF3an ---> 0
Rt.C.: prot_phosph_degr_r

***** Right arm recognition of dsRNA and TLR3_TRIF_TRAF6 complex formation *****

------ dsRNA degradation induced by TLR3i:
Ch.E.: TLR3i + dsRNA ---> TLR3i
Rt.C.: dsRNA_degr_r / prot_avg

------ TLR3i activation:
Ch.E.: dsRNA + TLR3i ---> dsRNA + TLR3a
Rt.C.: dsRNA_recogn_r / prot_avg

------ TLR3a degradation:
Ch.E.: TLR3a ---> 0
Rt.C.: prot_phosph_degr_r

------ TLR3a and TRIF binding:
Ch.E.: TLR3a + TRIF ---> TLR3_TRIF
Rt.C.: prot_bind_r / prot_avg

------ TLR3_TRIF degradation:
Ch.E.: TLR3_TRIF ---> 0
Rt.C.: prot_phosph_degr_r

------ TLR3_TRIF and TRAF6 binding:
Ch.E.: TLR3_TRIF + TRAF6 ---> TLR3_TRIF_TRAF6
Rt.C.: prot_bind_r / prot_avg

------ TLR3_TRIF_TRAF6 degradation:
Ch.E.: TLR3_TRIF_TRAF6 ---> 0
Rt.C.: prot_phosph_degr_r

***** TLR3dTF activation by TLR3_TRIF_TRAF6 *****

------ TLR3dTFi activation:
Ch.E.: TLR3_TRIF_TRAF6 + TLR3dTFi ---> TLR3_TRIF_TRAF6 + TLR3dTFa
Rt.C.: prot_activ_r / prot_avg

------ TLR3dTFa import:
Ch.E.: TLR3dTFa ---> TLR3dTFan
Rt.C.: prot_import_r

------ TLR3dTFan degradation:
Ch.E.: TLR3dTFan ---> 0
Rt.C.: prot_phosph_degr_r / 2

******* IKK2 (IKKg/IKKa/IKKb) activation by TLR3_TRIF_TRAF6 and regulation by A20 *******

------ IKK2i activation:
Ch.E.: TLR3_TRIF_TRAF6 + IKK2i ---> TLR3_TRIF_TRAF6 + IKK2a
Rt.C.: prot_activ_r / prot_avg

------ IKK2a degradation:
Ch.E.: IKK2a ---> 0
Rt.C.: prot_phosph_degr_r

------ IKK2a degradation due to A20:
Ch.E.: A20 + IKK2a ---> A20
Rt.C.: prot_phosph_degr_r / prot_avg / 2

******* IKK2 degradation of IkBa, RelA nuclear translocation, RelA_IkBa binding *******

------ IkBa degradation due to IKK2a:
Ch.E.: IKK2a + IkBa ---> IKK2a
Rt.C.: prot_phosph_degr_r / prot_avg * 1000

------ RelA_IkBa degradation:
Ch.E.: RelA_IkBa ---> 0
Rt.C.: prot_degr_r

------ RelA dissociation (and degradation of IkBa) due to IKK2a:
Ch.E.: IKK2a + RelA_IkBa ---> IKK2a + RelA
Rt.C.: prot_phosph_degr_r / prot_avg * 1000

------ RelA and IkBa binding:
Ch.E.: RelA + IkBa ---> RelA_IkBa
Rt.C.: prot_bind_r / prot_avg

------ RelA_IkBa spontaneous dissociation:
Ch.E.: RelA_IkBa ---> RelA + IkBa
Rt.C.: prot_diss_r

------ RelA nuclear import:
Ch.E.: RelA ---> RelAn
Rt.C.: prot_import_r

------ RelAn and IkBan binding:
Ch.E.: RelAn + IkBan ---> RelAn_IkBan

Rt.C.: cyto_to_nuc_ratio * prot_bind_r / prot_avg

------ RelAn_IkBan degradation:
Ch.E.: RelAn_IkBan ---> 0
Rt.C.: prot_degr_r

------ RelA_IkBa import:
Ch.E.: RelA_IkBa ---> RelAn_IkBan
Rt.C.: prot_import_r / 10

------ RelAn_IkBan export:
Ch.E.: RelAn_IkBan ---> RelA_IkBa
Rt.C.: prot_export_r

******* IRF7 dependent genes *******

******* RIGI production and degradation *******

------ RIGIg activation:
Ch.E.: IRF7an + RIGIg ---> RIGIg_IRF7an
Rt.C.: gene_act_r / prot_avg / 500 * 10

------ RIGIg_IRF7an inactivation:
Ch.E.: RIGIg_IRF7ab ---> RIGIg + IRF7an
Rt.C.: gene_inact_r / 500

------ RIGIt transcription:
Ch.E.: RIGIg_IRF7an ---> RIGIg_IRF7an + RIGIt
Rt.C.: mRNA_transc_r * 2

------ RIGIt basal transcription:
Ch.E.: RIGIg ---> RIGIg + RIGIt
Rt.C.: mRNA_basal_transc_r

------ RIGIt degradation:
Ch.E.: RIGIt ---> 0
Rt.C.: ifelse(RIGIt_siRNA, 8 * siRNA_incr_degr_r, 1) * mRNA_degr_r

------ RIGI synthesis:
Ch.E.: RIGIt ---> RIGIt + RIGI
Rt.C.: prot_transl_r

------ RIGI degradation:
Ch.E.: RIGI ---> 0
Rt.C.: prot_degr_r

******* TLR3dTF dependent genes *******

******* MAVS production and degradation *******

------ MAVSg activation:
Ch.E.: TLR3dTFan + MAVSg ---> MAVSg_TLR3dTFan
Rt.C.: gene_act_r / prot_avg / 100

------ MAVSg_TLR3dTFan inactivation:
Ch.E.: MAVSg_TLR3dTFab ---> MAVSg + TLR3dTFan
Rt.C.: gene_inact_r / 100

------ MAVSt transcription:
Ch.E.: MAVSg_TLR3dTFan ---> MAVSg_TLR3dTFan + MAVSt
Rt.C.: mRNA_transc_r * 1.5

------ MAVSt basal transcription:
Ch.E.: MAVSg ---> MAVSg + MAVSt
Rt.C.: mRNA_basal_transc_r

------ MAVSt degradation:
Ch.E.: MAVSt ---> 0
Rt.C.: mRNA_degr_r

------ MAVS synthesis:
Ch.E.: MAVSt ---> MAVSt + MAVS
Rt.C.: prot_transl_r

------ MAVS degradation:
Ch.E.: MAVS ---> 0
Rt.C.: prot_degr_r

******* IKK1 (TBK1/IKKe) production and degradation *******

------ IKK1g activation:
Ch.E.: TLR3dTFan + IKK1g ---> IKK1g_TLR3dTFan
Rt.C.: gene_act_r / prot_avg * 3

------ IKK1g_TLR3dTFan inactivation:
Ch.E.: IKK1g_TLR3dTFab ---> IKK1g + TLR3dTFan
Rt.C.: gene_inact_r

------ IKK1t transcription:
Ch.E.: IKK1g_TLR3dTFan ---> IKK1g_TLR3dTFan + IKK1t
Rt.C.: mRNA_transc_r

------ IKK1t basal transcription:

Ch.E.: IKK1g ---> IKK1g + IKK1t
Rt.C.: mRNA_basal_transc_r

------ IKK1t degradation:
Ch.E.: IKK1t ---> 0
Rt.C.: mRNA_degr_r

------ IKK1i synthesis:
Ch.E.: IKK1t ---> IKK1t + IKK1i
Rt.C.: prot_transl_r

------ IKK1i degradation:
Ch.E.: IKK1i ---> 0
Rt.C.: prot_degr_r

******* IKK2 (IKKg/IKKa/IKKb) production and degradation *******

------ IKK2g activation:
Ch.E.: TLR3dTFan + IKK2g ---> IKK2g_TLR3dTFan
Rt.C.: gene_act_r / prot_avg * 3

------ IKK2g_TLR3dTFan inactivation:
Ch.E.: IKK2g_TLR3dTFab ---> IKK2g + TLR3dTFan
Rt.C.: gene_inact_r

------ IKK2t transcription:
Ch.E.: IKK2g_TLR3dTFan ---> IKK2g_TLR3dTFan + IKK2t
Rt.C.: mRNA_transc_r

------ IKK2t basal transcription:
Ch.E.: IKK2g ---> IKK2g + IKK2t
Rt.C.: mRNA_basal_transc_r

------ IKK2t degradation:
Ch.E.: IKK2t ---> 0
Rt.C.: ifelse(IKK2t_siRNA, 0.6 * siRNA_incr_degr_r, 1) * mRNA_degr_r

------ IKK2i synthesis:
Ch.E.: IKK2t ---> IKK2t + IKK2i
Rt.C.: prot_transl_r

------ IKK2i degradation:
Ch.E.: IKK2i ---> 0
Rt.C.: prot_degr_r

******* IRF3 production and degradation, inhibition by RelA *******

------ IRF3g inhibition:

Ch.E.: RelAn + IRF3g ---> IRF3g_RelAn
Rt.C.: gene_act_r / prot_avg * 5

------ IRF3g_RelAn inhibition release:
Ch.E.: IRF3g_RelA ---> IRF3g + RelAn
Rt.C.: gene_inact_r

------ IRF3g activation:
Ch.E.: TLR3dTFan + IRF3g ---> IRF3g_TLR3dTFan
Rt.C.: gene_act_r / prot_avg * 3

------ IRF3g_TLR3dTFan inactivation:
Ch.E.: IRF3g_TLR3dTFan ---> IRF3g + TLR3dTFan
Rt.C.: gene_inact_r

------ IRF3t transcription:
Ch.E.: IRF3g_TLR3dTFan ---> IRF3g_TLR3dTFan + IRF3t
Rt.C.: mRNA_transc_r

------ IRF3t basal transcription:
Ch.E.: IRF3g ---> IRF3g + IRF3t
Rt.C.: mRNA_basal_transc_r

------ IRF3t degradation:
Ch.E.: IRF3t ---> 0
Rt.C.: ifelse(IRF3t_siRNA, 0.50 * siRNA_incr_degr_r, 1) * mRNA_degr_r

------ IRF3ii synthesis:
Ch.E.: IRF3t ---> IRF3t + IRF3ii
Rt.C.: prot_transl_r

------ IRF3ii degradation:
Ch.E.: IRF3ii ---> 0
Rt.C.: prot_degr_r

------ IRF3ii to IRF3i:
Ch.E.: IRF3ii ---> IRF3i
Rt.C.: prot_transition_r

------ IRF3i degradation:
Ch.E.: IRF3i ---> 0
Rt.C.: prot_degr_r

------ IRF3i import:
Ch.E.: IRF3i ---> IRF3in
Rt.C.: prot_import_r / 10

------ IRF3in export:
Ch.E.: IRF3in ---> IRF3i
Rt.C.: prot_export_r

------ IRF3in degradation:
Ch.E.: IRF3in ---> 0
Rt.C.: prot_degr_r

******* RelA production and degradation, inhibition by IRF3 *******

------ RelAg inhibition by IRF3an:
Ch.E.: IRF3an + RelAg ---> RelAg_IRF3an
Rt.C.: gene_act_r / prot_avg * RelAn_IRF3an_ratio

------ RelAg_IRF3an inhibition release:
Ch.E.: RelAg_IRF3ab ---> RelAg + IRF3an
Rt.C.: gene_inact_r / 10

------ RelAg activation:
Ch.E.: TLR3dTFan + RelAg ---> RelAg_TLR3dTFan
Rt.C.: gene_act_r / prot_avg

------ RelAg_TLR3dTFan inactivation:
Ch.E.: RelAg_TLR3dTFab ---> RelAg + TLR3dTFan
Rt.C.: gene_inact_r

------ RelAt transcription:
Ch.E.: RelAg_TLR3dTFan ---> RelAg_TLR3dTFan + RelAt
Rt.C.: mRNA_transc_r

------ RelAt basal transcription:
Ch.E.: RelAg ---> RelAg + RelAt
Rt.C.: mRNA_basal_transc_r

------ RelAt degradation:
Ch.E.: RelAt ---> 0
Rt.C.: ifelse(RelAt_siRNA, 1 * siRNA_incr_degr_r, 1) * mRNA_degr_r

------ RelAi synthesis:
Ch.E.: RelAt ---> RelAt + RelAi
Rt.C.: prot_transl_r

------ RelAi degradation:
Ch.E.: RelAi ---> 0
Rt.C.: prot_degr_r

------ RelAi to RelA:
Ch.E.: RelAi ---> RelA
Rt.C.: prot_transition_r / 10

------ RelA to RelAi:
Ch.E.: RelA ---> RelAi

Rt.C.: prot_transition_r / 10

------ RelAi to RelA due to IKK2a:
Ch.E.: IKK2a + RelAi ---> IKK2a + RelA
Rt.C.: prot_transition_r / prot_avg

------ RelA degradation:
Ch.E.: RelA ---> 0
Rt.C.: prot_degr_r

------ RelAn degradation:
Ch.E.: RelAn ---> 0
Rt.C.: prot_degr_r

******* RelA dependent genes *******

******* A20 production and degradation *******

------ A20g activation:
Ch.E.: RelAn + A20g ---> A20g_RelAn
Rt.C.: gene_act_r / prot_avg * 2 / 25

------ A20g_RelAn inactivation:
Ch.E.: IkBan + A20g_RelAn ---> A20g + RelAn_IkBan
Rt.C.: gene_inact_r / prot_avg * 2

------ A20t transcription:
Ch.E.: A20g_RelAn ---> A20g_RelAn + A20t
Rt.C.: mRNA_transc_r

------ A20t degradation:
Ch.E.: A20t ---> 0
Rt.C.: ifelse(A20t_siRNA, 6.5 * siRNA_incr_degr_r, 1) * mRNA_degr_r

------ A20 synthesis:
Ch.E.: A20t ---> A20t + A20
Rt.C.: prot_transl_r

------ A20 degradation:
Ch.E.: A20 ---> 0
Rt.C.: prot_degr_r

******* IkBa production and degradation *******

------ IkBag activation:
Ch.E.: RelAn + IkBag ---> IkBag_RelAn
Rt.C.: gene_act_r / prot_avg / 4

------ IkBag_RelAn inactivation:
Ch.E.: IkBan + IkBag_RelAb ---> IkBag + RelAn_IkBan
Rt.C.: gene_inact_r / prot_avg / 2 * 5

------ IkBat transcription:
Ch.E.: IkBag_RelAn ---> IkBag_RelAn + IkBat
Rt.C.: mRNA_transc_r * 5

------ IkBat degradation:
Ch.E.: IkBat ---> 0
Rt.C.: mRNA_degr_r * 4

------ IkBa synthesis:
Ch.E.: IkBat ---> IkBat + IkBa
Rt.C.: prot_transl_r * 10

------ IkBa degradation:
Ch.E.: IkBa ---> 0
Rt.C.: prot_degr_r * 10

------ IkBa import:
Ch.E.: IkBa ---> IkBan
Rt.C.: prot_import_r

------ IkBan degradation:
Ch.E.: IkBan ---> 0
Rt.C.: prot_degr_r * 10

------ IkBan export:
Ch.E.: IkBan ---> IkBa
Rt.C.: prot_export_r / 2

******* IRF7 production and degradation *******

#NAME?
Ch.E.: RelAn + IRF7g ---> IRF7g_RelAn
Rt.C.: gene_act_r / prot_avg * 3

------ IRF7g_RelAn inactivation:
Ch.E.: IRF7g_RelAn ---> IRF7g + RelAn
Rt.C.: gene_inact_r

------ IRF7t transcription:
Ch.E.: IRF7g_RelAn ---> IRF7g_RelAn + IRF7t
Rt.C.: mRNA_transc_r

------ IRF7t degradation:
Ch.E.: IRF7t ---> 0
Rt.C.: ifelse(IRF7t_siRNA, 0.50 * siRNA_incr_degr_r, 1) * mRNA_degr_r

------ IRF7a synthesis:
Ch.E.: IRF7t ---> IRF7t + IRF7a
Rt.C.: prot_transl_r

------ IRF7a import:
Ch.E.: IRF7a ---> IRF7an
Rt.C.: prot_import_r

------ IRF7an degradation:
Ch.E.: IRF7an ---> 0
Rt.C.: prot_phosph_degr_r / 2

******* IRF3 dependent genes *******

******* ISG56 production and degradation *******

------ ISG56g activation:
Ch.E.: IRF3an + ISG56g ---> ISG56g_IRF3an
Rt.C.: gene_act_r / prot_avg * RelAn_IRF3an_ratio * 5

------ ISG56g_IRF3an inactivation:
Ch.E.: ISG56g_IRF3ab ---> ISG56g + IRF3an
Rt.C.: gene_inact_r

------ ISG56t transcription:
Ch.E.: ISG56g_IRF3an ---> ISG56g_IRF3an + ISG56t
Rt.C.: mRNA_transc_r

------ ISG56t degradation:
Ch.E.: ISG56t ---> 0
Rt.C.: mRNA_degr_r

------ ISG56 synthesis:
Ch.E.: ISG56t ---> ISG56t + ISG56
Rt.C.: prot_transl_r

------ ISG56 degradation:
Ch.E.: ISG56 ---> 0
Rt.C.: prot_degr_r

******* RelA/IRF3 dependent genes *******

******* IFNb production and degradation *******

------ IFNbg activation by RelAn:
Ch.E.: RelAn + IFNbg ---> IFNbg_RelAn
Rt.C.: gene_act_r / prot_avg / 100 * 75

------ IFNbg_RelAn inactivation:
Ch.E.: IkBan + IFNbg_RelAn ---> IFNbg + RelAn_IkBan
Rt.C.: gene_inact_r / prot_avg * 20

------ IFNbg_RelAn activation by IRF3an:
Ch.E.: IRF3an + IFNbg_RelAn ---> IFNbg_RelAn_IRF3an
Rt.C.: gene_act_r / prot_avg * RelAn_IRF3an_ratio / 100

------ IFNbg_RelAn_IRF3an inactivation:
Ch.E.: IFNbg_RelAn_IRF3an ---> IFNbg + IRF3an + RelAn
Rt.C.: gene_inact_r / 1000

------ IFNbt transcription by IFNbg_RelAn_IRF3an:
Ch.E.: IFNbg_RelAn_IRF3an ---> IFNbg_RelAn_IRF3an + IFNbt
Rt.C.: mRNA_transc_r * 5

------ IFNbt degradation:
Ch.E.: IFNbt ---> 0
Rt.C.: mRNA_degr_r

------ IFNb synthesis:
Ch.E.: IFNbt ---> IFNbt + IFNb
Rt.C.: prot_transl_r

------ IFNb degradation:
Ch.E.: IFNb ---> 0
Rt.C.: prot_degr_r

******* Unregulated genes *******

******* TLR3 production and degradation *******

------ TLR3t basal transcription:
Ch.E.: TLR3g ---> TLR3g + TLR3t
Rt.C.: mRNA_basal_transc_r

------ TLR3t degradation:
Ch.E.: TLR3t ---> 0
Rt.C.: mRNA_degr_r

------ TLR3i synthesis:
Ch.E.: TLR3t ---> TLR3t + TLR3i
Rt.C.: prot_unreg_transl_r

------ TLR3i degradation:
Ch.E.: TLR3i ---> 0
Rt.C.: prot_unreg_degr_r

******* TRAF3 production and degradation *******

------ TRAF3t basal transcription:
Ch.E.: TRAF3g ---> TRAF3g + TRAF3t
Rt.C.: mRNA_basal_transc_r

------ TRAF3t degradation:
Ch.E.: TRAF3t ---> 0
Rt.C.: mRNA_degr_r

------ TRAF3 synthesis:
Ch.E.: TRAF3t ---> TRAF3t + TRAF3
Rt.C.: prot_unreg_transl_r

------ TRAF3 degradation:
Ch.E.: TRAF3 ---> 0
Rt.C.: prot_unreg_degr_r

******* TRAF6 production and degradation *******

------ TRAF6t basal transcription:
Ch.E.: TRAF6g ---> TRAF6g + TRAF6t
Rt.C.: mRNA_basal_transc_r

------ TRAF6t degradation:
Ch.E.: TRAF6t ---> 0
Rt.C.: mRNA_degr_r

------ TRAF6 synthesis:
Ch.E.: TRAF6t ---> TRAF6t + TRAF6
Rt.C.: prot_unreg_transl_r

------ TRAF6 degradation:
Ch.E.: TRAF6 ---> 0
Rt.C.: prot_unreg_degr_r

******* TRIF production and degradation *******

------ TRIFt basal transcription:
Ch.E.: TRIFg ---> TRIFg + TRIFt
Rt.C.: mRNA_basal_transc_r

------ TRIFt degradation:
Ch.E.: TRIFt ---> 0
Rt.C.: mRNA_degr_r

------ TRIF synthesis:
Ch.E.: TRIFt ---> TRIFt + TRIF

Rt.C.: prot_unreg_transl_r

------ TRIF degradation:
Ch.E.: TRIF ---> 0
Rt.C.: prot_unreg_degr_r

******* TLR3dTF production and degradation *******

------ TLR3dTFt basal transcription:
Ch.E.: TLR3dTFg ---> TLR3dTFg + TLR3dTFt
Rt.C.: mRNA_basal_transc_r

------ TLR3dTFt degradation:
Ch.E.: TLR3dTFt ---> 0
Rt.C.: mRNA_degr_r

------ TLR3dTFi synthesis:
Ch.E.: TLR3dTFt ---> TLR3dTFt + TLR3dTFi
Rt.C.: prot_unreg_transl_r

------ TLR3dTFi degradation:
Ch.E.: TLR3dTFi ---> 0
Rt.C.: prot_unreg_degr_r

******* End of model definition *******

Supplemental Table ST4: TFBS analysis. Table presents counts of TFBS corresponding to IRF family and NF-κB family transcription factors found in promoters of presented genes in four species: cattle (bosTau), mouse (mm), chimpanzee (panTro) and human (hg). Yellow rows correspond to promoter sequences with at least one IRF3 binding site, bright orange rows correspond to sequences containing motifs for all 3 members of IRF family, red cells correspond to sequences with more than 2 binding sites for IRF3. For genes: IRF7, MAVS, IKK1, IKBKB and DDX58 only human and murine promoters were analyzed.

Query ID	RefSeq ID	IRF1	IRF2	IRF3	IRF7	NF-kappaB	NFKB1	REL	RELA	AP1	SP1
IRF1-b	bosTau7_refGene_NM_001191261	2	0	0	0	5	5	10	7	3	6
IRF1-m	mm9_knownGene_uc007iww.2	0	0	0	0	4	2	7	5	8	4
IRF1-m	mm9_knownGene_uc007iwz.2	0	0	0	0	4	3	7	5	7	4
IRF1-c	panTro gi|291061371	1	0	0	0	4	5	9	6	5	12
IRF1-h	hg19_knownGene_uc003kxa.2	1	0	0	0	4	5	9	6	5	11
IRF1-h	hg19_knownGene_uc003kxb.2	2	0	0	1	5	2	3	2	7	12
IRF1-h	hg19_knownGene_uc010jdt.2	0	0	0	2	2	0	2	1	6	6
IRF2-b	bosTau7_refGene_NM_001205793	3	1	1	2	5	2	6	2	9	9
IRF2-m	mm9_knownGene_uc009lqo.2	3	1	1	1	6	3	6	3	4	13
IRF2-m	mm9_knownGene_uc009lqp.1	1	0	0	1	2	0	6	1	12	2
IRF2-c	panTro gi|291061372	2	1	1	0	1	1	7	1	6	2
IRF2-h	hg19_knownGene_uc003iwf.4	2	1	1	2	2	1	4	1	10	11
IRF3-b	bosTau7_refGene_NM_001029845	1	0	1	1	3	1	4	2	11	10
IRF3-m	mm9_knownGene_uc009gsm.1	1	0	1	1	4	1	5	3	12	5
IRF3-m	mm9_knownGene_uc009gsn.1	1	0	1	1	4	1	5	3	12	5
IRF3-m	mm9_knownGene_uc012fka.1	2	0	1	1	4	1	3	2	9	2
IRF3-c	panTro gi|291061357	1	0	1	1	1	0	4	0	4	8
IRF3-h	hg19_knownGene_uc010end.2	0	0	1	0	1	0	4	0	5	8
IRF3-h	hg19_knownGene_uc002poz.1	1	0	2	1	1	0	4	0	4	8
IRF3-h	hg19_knownGene_uc010ene.1	1	0	0	1	3	2	5	2	4	0
IRF7-mv1	mm10_knownGene_uc009kkg.2	1	0	0	1	3	0	5	3	15	5
IRF7-mv2	mm10_knownGene_uc012fwt.2	1	0	0	2	3	0	4	3	13	6
IRF7-hv1	hg19_knownGene_uc001lqf.3	4	2	2	2	1	2	6	1	6	10
IRF7-hv2	hg19_knownGene_uc001lqg.3	5	2	2	2	1	2	5	1	7	10
IRF7-hv3	hg19_knownGene_uc001lqh.3	5	2	2	2	0	0	3	0	10	10
IRF7-hv4	hg19_knownGene_uc009ycb.3	1	1	1	0	1	2	5	1	7	16
NFKB1-b	bosTau7_refGene_NM_001076409	4	0	0	1	0	0	2	0	11	2
NFKB1-m	mm9_knownGene_uc008rlw.1	1	0	0	1	0	1	2	0	8	3
NFKB1-m	mm9_knownGene_uc008rlx.1	1	0	0	1	4	3	8	3	7	3
NFKB1-m	mm9_knownGene_uc012cye.1	2	0	1	2	3	1	5	3	14	3
NFKB1-m	mm9_knownGene_uc012cyf.1	0	0	0	0	1	2	5	1	14	5
NFKB1-m	mm9_knownGene_uc012cyg.1	1	0	0	0	0	0	5	0	14	3
NFKB1-c	panTro3_xenoRefGene_NM_001165412	2	0	2	2	3	3	3	4	7	5
NFKB1-h	hg19_refGene_NM_001165412	3	0	2	3	4	3	3	3	10	8
NFKB2-b	bosTau7_refGene_NM_001102101	0	0	0	0	5	4	5	2	6	10
NFKB2-m	mm9_knownGene_uc008hst.2	0	0	0	0	4	2	6	4	12	10
NFKB2-m	mm9_knownGene_uc008hsv.1	0	0	0	0	4	2	6	4	12	10
NFKB2-m	mm9_knownGene_uc008hsx.2	0	0	1	0	6	5	7	4	11	9
NFKB2-m	mm9_knownGene_uc008hsy.2	0	0	1	0	5	5	7	3	10	15
NFKB2-c	panTro3_gold_AACZ03073940.1	0	0	0	0	6	4	5	3	6	10
NFKB2-h	hg19_knownGene_uc001kva.3	1	0	0	0	2	6	3	2	7	8
NFKB2-h	hg19_knownGene_uc001kvb.3	1	0	0	0	4	8	4	4	4	10
NFKB2-h	hg19_knownGene_uc001kvd.3	0	0	0	0	6	4	5	3	6	10
NFKB2-h	hg19_knownGene_uc009xxc.3	0	0	0	0	7	5	7	4	6	12
NFKBIA-b	bosTau7_refGene_NM_001045868	2	1	0	1	6	5	11	4	7	8
NFKBIA-m	mm9_refGene_NM_010907	3	0	1	3	6	4	6	5	6	6
NFKBIA-c	panTro gi|291061362	1	0	0	0	1	0	2	0	7	3
NFKBIA-h	hg19_knownGene_uc001wtf.4	2	1	1	3	7	4	5	3	2	7
NFKBIE-b	bosTau7_refGene_NM_001130746	3	1	3	2	5	4	5	3	13	6
NFKBIE-m	mm9_knownGene_uc008cqv.1	0	0	1	1	3	3	4	2	17	5
NFKBIE-c	panTro gi|291061370	5	1	4	4	3	3	3	2	13	5
NFKBIE-h	hg19_knownGene_uc003oxe.1	5	1	4	4	3	3	3	2	13	5
RELA-b	bosTau7_refGene_NM_001080242	0	0	0	1	1	2	3	1	5	6
RELA-m	mm9_refGene_NM_009045	1	0	1	3	3	3	5	2	4	4
RELA-c	panTro3_refGene_NM_001246630	1	0	0	0	1	1	1	2	7	7
RELA-h	hg19_refGene_NM_001145138	1	0	0	1	4	2	4	3	5	11
REL-b	bosTau7_refGene_NM_001192970	0	0	0	0	1	1	1	0	15	1
REL-m	mm9_refGene_NM_009044	0	0	0	2	4	4	5	4	4	7
REL-c	panTro3_gold_AACZ03012369.1	0	0	0	1	3	4	2	2	4	15
REL-h	hg19_refGene_NM_002908	0	0	0	1	4	4	3	3	4	17
Mavs-m	mm10_knownGene_uc008mld.2	1	0	0	2	1	1	4	1	8	15
MAVS-h	hg19_knownGene_uc002wjv.3	2	0	0	1	3	1	3	2	11	28
Ikk1-m	mm10_knownGene_uc008hpg.2	0	0	0	2	1	1	4	2	11	2
IKK1-h	hg19_knownGene_uc001kqp.3	1	0	0	1	4	1	1	1	10	3
IKBKB-hv1	hg19_knownGene_uc003xov.3	1	0	0	1	3	5	4	1	10	8
IKBKB-hv2	hg19_knownGene_uc022auj.1	0	0	0	2	4	5	5	4	9	6

Query ID	RefSeq ID	IRF1	IRF2	IRF3	IRF7	NF-kappaB	NFKB1	REL	RELA	AP1	SP1
Ikbkb-mv1	mm10_knownGene_uc009ldm.2	1	0	0	0	1	0	3	1	7	9
Ikbkb-mv2	mm10_knownGene_uc009ldq.1	0	0	0	0	2	2	4	1	6	20
DDX58-hv1	hg19_knownGene_uc003zra.3	9	5	3	12	2	1	3	2	12	8
DDX58-hv2	hg19_knownGene_uc010mji.3	0	1	0	1	1	1	2	0	10	4
Ddx58-mv1	mm10_knownGene_uc008she.1	2	2	0	6	2	2	10	1	13	10
Ddx58-mv2	mm10_knownGene_uc008shf.1	2	1	1	2	2	0	3	2	5	53

Table ST5: Effect of siRNA knockdown on dsRNA-induced NF-kB/IRF3 gene expressions in A549 cells. Statistical significance of the difference recorded in the knockdown experiment, carried out using the 2-sample, 2-sided t-test (Welch test), corresponding to the bar charts in Figure 3. (A) Comparison of the mRNA-specific siRNA knockdown versus control (nonspecific siRNA), in dsRNA-nonstimulated and dsRNA-stimulated experiment (at 6hr). (B) Comparison of dsRNA-induced versus dsRNA-noniduced under siRNA knockdown (at 6hr). Rows: Different knockdowns. Columns: Genes expressed.

*Minus sign at the p-value denotes reduced expression. For example, -0.002 denotes p = 0.002 and reduced expression a a result of knockdown (panel A) or as result of dsRNA induction (panel B).

(A) Comparison of control (at 0 hr) and knockdown (at 6 hr)

	Rel A	IRF3	RIG-I	Ikkg	A20	IkBa	ISG56	IFN
**RelAsiRNA**	***-0.002***	*0.007*	*-0.039*	*0.017*	*-0.041*	-0.066	*-0.026*	-0.541
**RelAsiRNA+dsRNA**	***0.000***	*0.010*	*-0.006*	*0.009*	***-0.002***	***-0.001***	*0.008*	***-0.001***
**IRF3siRNA**	*0.011*	*-0.009*	-0.153	0.063	*0.045*	0.051	*-0.005*	*0.006*
**IRF3siRNA+dsRNA**	*0.014*	***-0.001***	-0.390	0.206	***0.000***	***0.003***	***0.000***	***0.000***
**RIGIsiRNA**	*0.012*	*0.012*	*-0.006*	*0.050*	0.182	-0.468	***-0.004***	*0.022*
**RIGsiRNA+dsRNA**	0.561	0.139	***0.000***	0.273	*0.043*	0.101	***0.000***	***0.000***
**IKKgsiRNA**	0.363	0.168	-0.098	***-0.001***	-0.140	0.608	*-0.006*	*0.021*
**IkkgsiRNA+dsRNA**	-0.107	0.154	*-0.011*	***-0.001***	*-0.008*	*-0.025*	*-0.042*	*-0.007*

(B) Comparison of dsRNA-induced to dsRNA-noniduced under siRNA knockdown (at 6hr)

	Rel A	IRF3	RIG-I	Ikkg	A20	IkBa	ISG56	IFN
**ConsiRNA**	***0.002***	***0.001***	***0.000***	-0.399	***0.001***	***0.001***	***0.000***	***0.000***
**RelAsiRNA**	0.756	***0.001***	***0.000***	0.799	***0.003***	*0.007*	***0.000***	***0.000***
**IRF3siRNA**	***0.004***	*0.021*	***0.000***	-0.182	***0.000***	***0.000***	***0.001***	***0.001***
**RIGIsiRNA**	*0.053*	***0.002***	***0.005***	*-0.038*	***0.000***	***0.000***	***0.002***	***0.001***
**IKKgsiRNA**	*0.022*	***0.001***	***0.000***	0.632	***0.002***	***0.001***	***0.000***	***0.000***

***p* -values**

italics, underscored ≤ 0.05

italics, boldface ≤ 0.01

p = 0.000 denotes p < 0.001

SF1: Wiring diagram corresponding to the pseudocode and differential equation system defining the mathematical model of innate immune response.

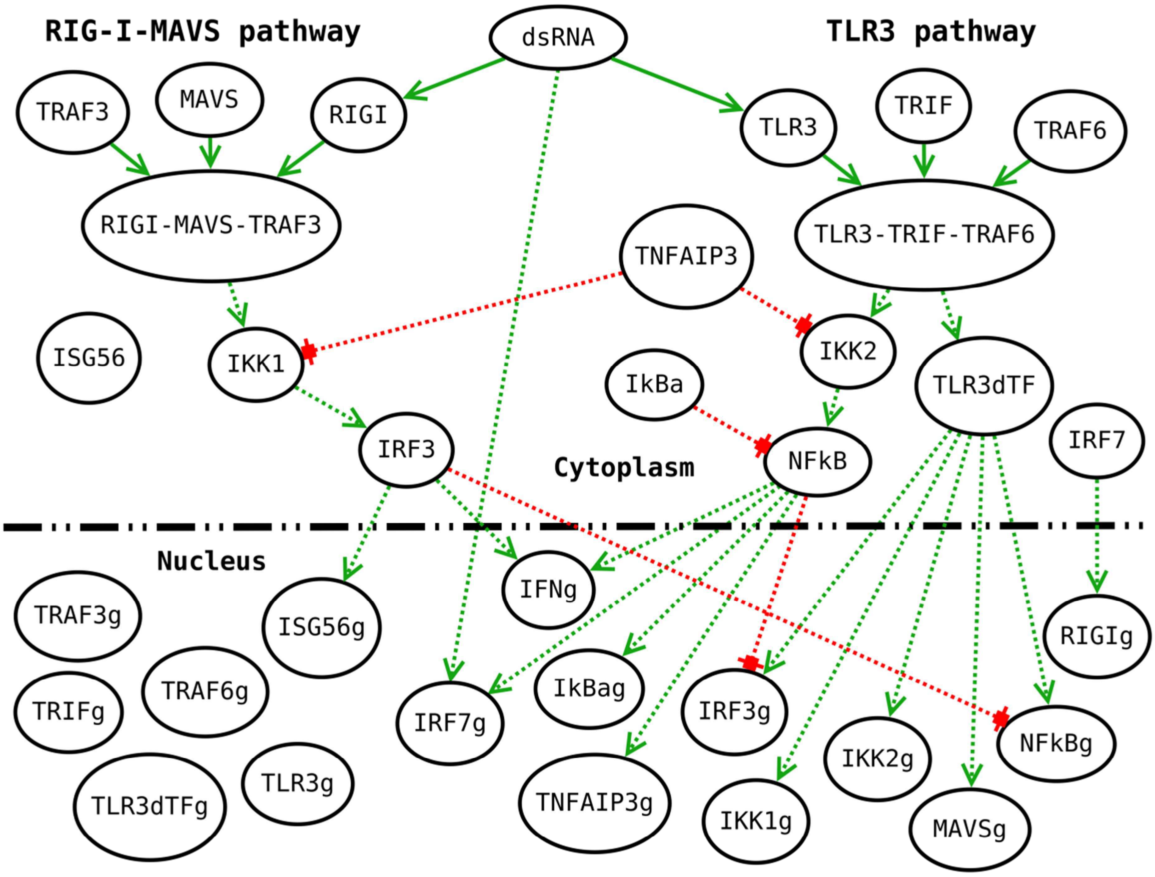

Notes

- Solid lines denote binding reactions
- Dot lines denote activation (green) or inhibition (red). The special case of a dotted line from dsRNA to IRF7g, means that NFkB only activates IRF7g in presence of dsRNA (in the differential equation system, this appears as I(dsRNA), meaning the indicator of dsRNA).
- We show only cytoplasmic proteins, without making difference if they are active or inactive. For example, there exist IRF3ii, IRF3i, and IRF3a, and we show IRF3. Translocation of the protein to nucleus is implicit in its action on a nuclear gene.
- We do not show the complex NFκB-IkBα
- We do not show mRNA

SF2: Snapshots of RelA-specific and IRF3-specific labeling in A549 cells at different times in non dsRNA-induced experiment.

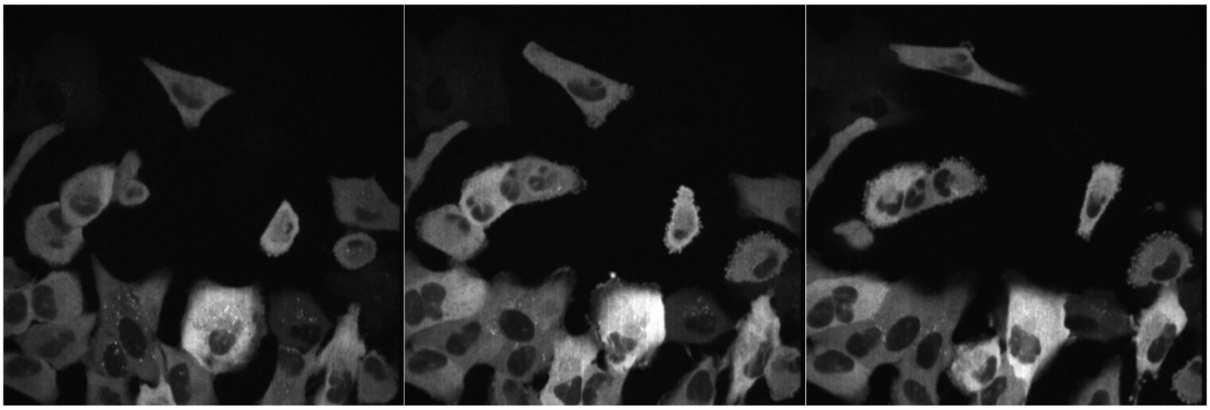

(A) RelA-specific labeling at times $t = 0$, 3 and 6 hr. No translocation from cytoplasm to nucleus has been observed.

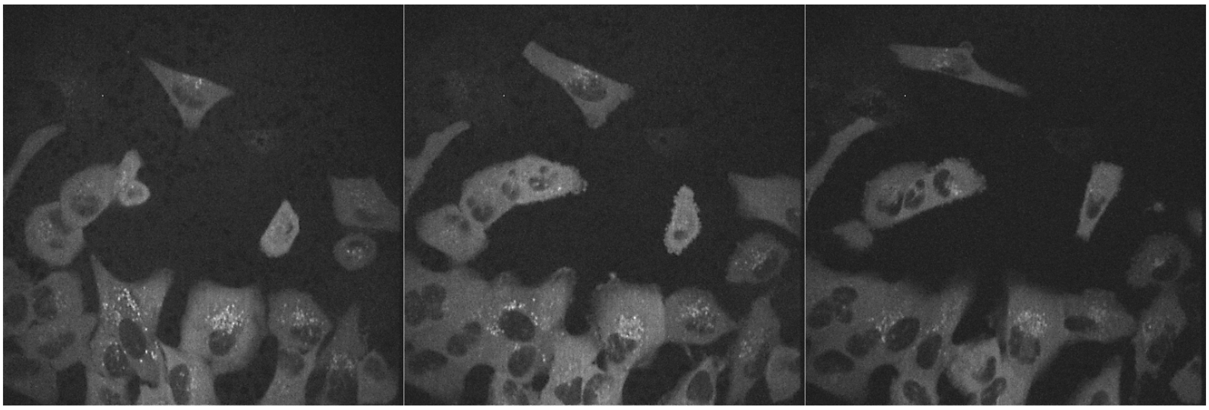

(B) IRF3-specific labeling at times $t = 0$, 3 and 6 hr. No translocation from cytoplasm to nucleus has been observed.
